# From RCT to mechanistic study: ATRA reverses myofibroblast activation by reprogramming glucose metabolism via HIC1 and PCK1/2 to attenuate hypertrophic scar formation

**DOI:** 10.1016/j.mmr.2026.100021

**Published:** 2026-04-27

**Authors:** Zi-Chao Li, Yi-Fu Zhu, Ya-Juan Song, Zhi-Jun Tan, Bin Liu, Yan Jiang, Hou-An Xiao, Dong-Mei Zu, Tong Wang, Yi Shi, Yan Jiao, Xue-Yong Li, Xing-Bo Xu, Lei Shang, Zhou Yu, Bao-Qiang Song

**Affiliations:** a Department of Plastic Surgery, Xijing Hospital, Fourth Military Medical University, Xi’an 710032, China; b Department of Plastic and Aesthetic Surgery, Peking Union Medical College Hospital, Chinese Academy of Medical Sciences & Peking Union Medical College, Beijing 100730, China; c Department of Health Statistics, Ministry of Education Key Laboratory of Hazard Assessment and Control in Special Operational Environment, School of Public Health, Fourth Military Medical University, Xi’an 710032, China; d Department of Burn, Plastic and Cosmetic Surgery, Xi’an Central Hospital, Xi’an Jiaotong University, Xi’an 710003, China; e Department of Cosmetology, the Second People’s Hospital of Jiangyou City, Jiangyou 621702, Sichuan, China; f Department of Burns, Plastic and Cosmetic Surgery, Xian Ninth Hospital, Xi’an 710054, China; g Department of Plastic Surgery, People’s Hospital of Ankang City, Ankang 725000, Shaanxi, China; h Department of Burn and Plastic Surgery, Tangdu Hospital, Fourth Military Medical University, Xi’an 710038, China; i Department of Burn and Plastic Surgery, the Second Affiliated Hospital of Xi’an Jiaotong University, Xi’an 710004, China; j Clinic for Cardiology and Pulmonology, University Medical Center Göttingen, Göttingen 37075, Germany

**Keywords:** All-trans retinoic acid (ATRA), Randomized controlled trial (RCT), Glucose metabolic reprogramming, Myofibroblast activation, Hypertrophic scar

## Abstract

**Background:**

Abnormal glucose metabolism often contributes to myofibroblast activation and the pathogenesis of skin fibrotic diseases. All-trans retinoic acid (ATRA), the active component of tretinoin cream, can regulate glucose metabolism and activate myofibroblasts. Importantly, investigating the potential of ATRA to inhibit myofibroblast activation by modulating glucose metabolism could reveal the translational significance of ATRA in attenuating hypertrophic scar (HS) formation.

**Methods:**

We first conducted a multicenter, double-blind, randomized controlled trial (RCT) to compare the effects of tretinoin cream with those of the first-line medication, silicone gel. In the mechanistic study, the characteristics of glucose metabolic reprogramming and the activation of hypertrophic scar fibroblasts (HSFs) after ATRA treatment were identified through multi-omics profiling, complemented by glucose metabolism assays and functional validations. Besides, genetic overexpression targeting the potential downstream molecules of ATRA, including hypermethylated in cancer 1 (HIC1), phosphoenolpyruvate carboxykinase (PCK)1, and PCK2, was conducted *in vitro* in HSFs and *in vivo* in skin fibroblasts of Col1a2-CreER mice.

**Results:**

Our RCT demonstrated that tretinoin cream is non-inferior to silicone gel in preventing HS formation, with the absolute risk difference of incidence rates [–8.65% 90% two-sided confidence interval (CI) –23.03 to 5.74] and in decreasing scar thickness [(2856.20±211.83) μm vs. (1664.57±273.50) μm], attributing to the reduction in HSF proliferation and the proportion of myofibroblasts. Moreover, tretinoin cream effectively mitigated HS formation in both mice and rabbits without impeding normal wound healing. Mechanistically, HSFs underwent glucose reprogramming, characterized by increased aerobic glycolysis, which facilitated the transition of HSFs to myofibroblasts and their proliferation. However, ATRA upregulated HIC1, PCK1, and PCK2 expression through retinoic acid receptor alpha (RARα) activation, thereby inhibiting the fibrotic phenotypes of HSFs by suppressing aerobic glycolysis and facilitating gluconeogenesis. The fibroblast-specific overexpression of HIC1, PCK1, or PCK2 in Col1a2-CreER mice significantly reduced myofibroblast activation and hypertrophic scarring.

**Conclusions:**

Our study not only substantiated that topical tretinoin cream could serve as an effective strategy to prevent HSs in clinical settings, but also established ATRA as a regulator of glucose metabolism. Importantly, ATRA/RARα-mediated glucose reprogramming was identified as a potential therapeutic target for attenuating HS formation.

**Trial registration:**

ChiCTR, ChiCTR2500097242. Registered on 14 Feb, 2025. Available from https://www.chictr.org.cn/bin/project/edit?pid=220146.

## Background

The excessive activation of myofibroblasts often contributes to the pathogenesis of hypertrophic scars (HSs), significantly impairing the aesthetic and functional properties of the skin and even leading to substantial physiological and psychological disorders for patients [1], [2]. Therefore, comprehending the mechanisms underlying myofibroblast activation and identifying drugs with translational potential to inhibit this process are crucial for mitigating HS pathogenesis in clinical practice. Currently, various invasive and non-invasive approaches are employed for the prevention and treatment of HSs, with silicone-based therapies recognized as the first-line option [3], [4]. Silicone-based products, including sheets and gels, are well-recognized for their efficacy in both HS and keloid management [3]. Nevertheless, despite silicone gel treatment, HSs still develop in over 30% of cases. Besides, the high cost of these products often deters patients from pursuing anti-scar therapies [5]. These limitations underscore the urgent need for affordable and effective alternatives for HS prevention and therapy.

A clinical study has suggested that topical tretinoin cream may serve as a promising candidate for inhibiting HS formation [6]. Currently, tretinoin cream, as well as its primary active ingredient, all-trans retinoic acid (ATRA), are extensively utilized in dermatology for the treatment of acne, psoriasis, and skin aging, demonstrating a well-established safety profile [7]. Further, mechanistic studies have demonstrated that ATRA in tretinoin cream can modulate collagen synthesis and degradation, thus facilitating scar remodeling, while the exogenous administration of ATRA typically exerts its effects through the activation of retinoic acid (RA) signaling [8], [9]. In addition, a recent study indicated that retinoic acid receptor (RAR)-dependent activation of RA signaling modulates fibroblast reprogramming, preventing the transition of fascia-derived fibroblasts into myofibroblasts, thereby potentially reducing scar formation *in vivo* [10]. Despite these promising findings, several limitations, such as the scarcity of large-scale prospective studies, the absence of well-designed double-blind randomized trials, and the heterogeneity in wound types treated with tretinoin, still impede the widespread clinical application of tretinoin cream [11], [12], [13]. Therefore, robust clinical evidence from randomized controlled trials (RCTs) is essential to definitively assess the efficacy and safety of tretinoin for HS prevention and treatment. Moreover, the precise molecular mechanisms by which ATRA modulates HS development remain to be fully elucidated.

Emerging evidence highlights that the Warburg effect, enhanced glycolytic activity coupled with reduced oxidative phosphorylation (OXPHOS) under aerobic conditions, contributes to abnormal wound healing [14]. Specifically, glycolysis-driven myofibroblast activation plays a pivotal role in skin-fibrosing disorders, such as keloid, which serves as an important pathogenic cause in HSs [15]. Mechanistically, heightened glycolysis activates fibrosis-associated signaling pathways such as transforming growth factor-β (TGF-β) signaling, thereby amplifying myofibroblast activation [16], [17]. Beyond glycolysis, impaired gluconeogenesis is implicated in hepatic and renal fibrosis and also influences the wound healing process [18], [19], [20]. Consequently, targeting glucose reprogramming may emerge as a promising therapeutic strategy for mitigating skin fibrosis. However, limited research has focused on the role of aerobic glycolysis and gluconeogenesis in myofibroblast activation during HS development, emphasizing the need for further studies.

To address these knowledge gaps, we first conducted a multicenter, double-blind RCT to evaluate the efficacy of topical tretinoin cream in attenuating HS formation compared to the first-line medication, silicone gel. Next, we investigated the impact of dysregulated glucose metabolism on myofibroblast activation and whether ATRA-induced metabolic reprogramming could attenuate HS development by modulating the fate of myofibroblasts. Particularly, the purpose of our study is to provide a comprehensive understanding of the role of ATRA in regulating myofibroblast fate and preventing HS formation.

## Materials and methods

### Clinical RCT study

#### Clinical trial design and participants

This study was conducted as a multi-center, double-blind, parallel, randomized controlled trial to evaluate the efficacy of topical silicone gel vs. 0.05% tretinoin cream in preventing HS formation following surgical incisions. The trial was conducted at three medical centers in China: Xijing Hospital (the primary sponsor), Xi’an Central Hospital, and Second People’s Hospital of Jiangyou City. The study adhered to the ethical principles outlined in the Declaration of Helsinki and used the CONSORT reporting guidelines [21], which received approval from the Ethics Committee of Xijing Hospital (KY20232367-F-1). Written informed consent was obtained from all participants prior to enrolment. The trial was registered with the Chinese Clinical Trial Registry (https://www.chictr.org.cn, ChiCTR2500097242). A total of 135 patients were initially recruited. After applying inclusion and exclusion criteria, 112 patients were included in the study. Inclusion criteria required participants to be aged 16–75 years with one or more full-thickness linear incisions on the neck, trunk, or extremities. Exclusion criteria included: 1) a history of severe systemic or malignant diseases; 2) diabetes mellitus; 3) skin fibrotic conditions such as keloids or scleroderma; 4) infected or contaminated incisions; 5) chronic wounds; 6) benign or malignant skin disorders affecting normal skin physiology (e.g., neurofibromas, psoriasis); 7) severe allergies to silicone or tretinoin; 8) pregnancy, lactation, or planned pregnancy; 9) concurrent use of other scar-preventive treatments (e.g., laser therapy); and 10) any condition deemed unsuitable by the investigators. The patients’ baseline information and follow-up evaluation forms were stored in the archives of the Department of Plastic Surgery at Xijing Hospital.

#### Randomization and blinding

A total of 112 patients enrolled at the three medical centers were randomly assigned to either the silicone group or the tretinoin group using a block randomization method. Randomization was computer-generated by an independent statistician. The study medication was prepared and packaged at Xijing Hospital by a clinical research coordinator (CRC) and placed in identical white aluminum tubes labeled with randomization numbers. Administration of the agent was conducted by an independent CRC at each medical center. Both investigators and participants remained blinded until the end of the study.

#### Interventions and procedures

The study procedure is illustrated in Additional file 1: Fig. S1. A total of 58 patients received topical silicone gel (Strataderm gel, Stratpharma, Switzerland), while 54 patients received topical 0.05% tretinoin cream (Tretinoin cream, Huapont, China). Both treatments were applied twice daily for 3 months. The topical agents were administered to postoperative incisions only after complete reepithelialization, which occurred 7 d after the stitches were removed. However, follow-up was not completed for 4 patients in the silicone group with 6 incisions and 2 patients in the tretinoin group with 6 incisions. If a patient experienced drug-related allergic reactions or intolerable side effects, the trial was terminated following the physician’s assessment. Standardized photographs were taken twice: once before treatment initiation and at 3 months after treatment. At the 3-month follow-up, the occurrence of HSs was assessed either in person at each medical center or via remote video consultation.

#### Outcomes and evaluations

The primary outcome was the occurrence of HSs, which was assessed by three experienced dermatologists not involved in the study based on standardized photographs obtained at the 3-month follow-up after treatment. Secondary outcomes included scar quality evaluation using the modified Vancouver Scar Scale (VSS) and the Patient and Observer Scar Assessment Scale (POSAS), assessment of treatment-related adverse events, skin ultrasound analysis, and histological examination of scars [22], [23]. The modified VSS, detailed in Additional file 1: Table S1, evaluates scar height, pliability, vascularity, and pigmentation. The POSAS questionnaire, as shown in Additional file 1: Table S2, consists of two scales: an observer scale (completed by investigators) that assesses vascularity, pigmentation, thickness, relief, pliability, and surface area, and a patient scale (completed by participants) that evaluates pain, itch, colour, stiffness, thickness, and regularity. The total scores from both assessments were used as an integrated measure of scar severity, with each item rated on a 10-point scale, where a score of 1 indicates normal skin and 10 represents the worst possible scar [23].

The side effects of the topical agents were assessed using the Medical Dictionary for Regulatory Activities (MedDRA). The system organ classes evaluated included general disorders and administration site conditions, infections and infestations, injury, poisoning and procedural complications, musculoskeletal and connective tissue disorders, nervous system disorders, and skin and subcutaneous tissue disorders [24]. We further assessed the graded severity (mild, moderate, and severe), duration time, remission (spontaneous remission, required intervention), and tolerance of the side effects (yes, no) following the topical application of tretinoin cream. In cases where a patient has multiple incisions, and one or more of them exhibit specific side effects following medication administration, the value corresponding to the incision exhibiting the highest severity grading, the longest duration, and the poorest tolerance should be selected as the characteristic value for the side effects following medication in this patient.

Of the 112 patients who participated in the RCT, 6 patients were lost to follow-up; only 106 participants completed a 3-month application of topical medications. From each group, 5 samples were randomly selected for skin ultrasound analysis using the LOGIQ E11 system (GE Healthcare, USA), based on their willingness to participate. The scar elevation index (SEI) was calculated using the formula: SEI=cross-sectional area of the entire scar/cross-sectional area below the scar ridge [25]. Besides, treatment failure was defined as a clinical diagnosis of HS formation. Upon patient request, HSs were excised for histological analysis, with three biological replicates performed for each group.

### *In vivo* study

#### Establishment of the Cre-loxP system

To activate Cre-recombinase, thirty Col1a2-CreER mice (Cyagen Biosciences Inc., China) received intraperitoneal injections of tamoxifen (CAS#10540-29-1, Sigma-Aldrich, USA) at a dose of 0.1 mg/g body weight for 5 consecutive days, starting 20 d before wound model construction. Tamoxifen was freshly prepared at a concentration of 10 mg/ml in corn oil (CAS#8001-30-7, Sigma-Aldrich, USA) according to established protocols [26]. To achieve targeted gene overexpression in skin fibroblasts, tamoxifen-induced 30 Col1a2-Cre mice (*n=*5 in each group) were intradermally and subcutaneously injected with 100 µl of adeno-associated virus (AAV) [10¹¹ genomic copies (GC)/ml] at and around the wound site. The injections were administered twice: 15 d before wound creation and 3 d post-operation. This approach facilitated the overexpression of *HIC1*, *PCK1*, and *PCK2* genes in skin fibroblasts. AAV serotype 9 was used for transfection in this study [27]. The following AAV constructs were utilized: AAV-CMV-loxP-stop-loxP-*HIC1* (AAV-*HIC1*), AAV-CMV-loxP-stop-loxP-*PCK1* (AAV-*PCK1*), AAV-CMV-loxP-stop-loxP-*PCK2* (AAV-*PCK2*), and AAV-CMV-loxP-stop-loxP (AAV-control). These constructs were generated using the triplasmid transfection method and purchased from Tsingke Biological Technology (TsingKe, China). In the absence of Cre recombinase, the target genes remained inactive due to the presence of a stop codon in the AAV construct. However, in fibroblasts expressing activated Cre recombinase, the stop codon was excised, allowing the target genes to be expressed efficiently. All animal experiments were approved by the Medical Ethics Committee of the Fourth Military Medical University (IACUC20241264).

#### Mouse wound splinting model

The wound splinting model was conducted with Balb/C mice and Col1a2-CreER mice, following previously established protocols [28]. Briefly, a full-thickness wound was created on the midline of the mouse back using an 8 mm biopsy punch after shaving and disinfecting the area. A silicone ring with an inner diameter of 8 mm was securely attached to the wound edge with glue to prevent wound contraction. For Balb/C mice (*n*=15), they were divided into 3 groups: Control group, Treat 1, and Treat 2. The wounds were treated with either PBS (Control group) or 0.05% tretinoin cream on day 0 (Treat 1) or day 10 post-operation (Treat 2). Wounds in both Balb/C and Col1a2-CreER mice were covered with sterile transparent films (Tegaderm, 3 M, USA). Photographs were taken on days 0, 4, 8, 12, 15, and 18 after wounding. The wound closure area was quantified using ImageJ software. Superficial blood flow in the healed wounds was assessed using Laser Speckle Contrast Imaging (LSCI) (RWD Life Science Co., Ltd., China) on day 18 postoperatively [29]. Skin tissue samples were collected for histological evaluation on the same day.

#### Rabbit ear HS model

HSs were induced on the ventral side of rabbit ears as previously described [30]. New Zealand White rabbits were obtained from the Experimental Animal Center of the Fourth Military Medical University. Briefly, 4 full-thickness circular skin resections (10 mm diameter) were excised down to the bare cartilage on each ear under sterile conditions. Six rabbits were divided into two groups: the control group and the Tretinoin group. After wound re-epithelialization on postoperative day 21, either 0.05% tretinoin cream or PBS was topically applied daily from days 21 to 55. Photographs were taken on days 0, 21, 28, 35, 42, 49, and 55. Superficial blood flow in the wounds was assessed using LSCI on day 55 post-surgery, after which tissue samples were harvested for histological evaluation.

#### Histological analysis of scar tissues

The wound healing and scar formation abilities were evaluated through histological analysis of excised scars obtained from human subjects, mouse dorsal skin, and rabbit ears. Hematoxylin and eosin (HE) staining was used to assess the general morphology of the scar tissue, while Masson’s trichrome staining was employed to evaluate collagen deposition. For tissue sections, immunofluorescence (IF) staining was performed overnight at 4 °C using primary antibodies targeting hypermethylated in cancer 1 (HIC1), PCK1, PCK2, and α-SMA (Additional file 1: Table S3). This was followed by incubation with fluorescently labeled secondary antibodies and DAPI for nuclear staining.

### *In vitro* study

#### Isolation and expansion of primary hypertrophic scar fibroblasts (HSFs)

HSFs were isolated from excised HS tissues obtained from 4 patients in the Xijing Hospital (Xi’an, Shaanxi, China). The patient information is provided in Additional file 1: Table S4. The study was approved by the Ethics Committee of Xijing Hospital (KY20232367-F-1). The isolation and culture of primary fibroblasts were conducted as previously described [30]. Briefly, the excised HS tissues were cleaned with sterile PBS (Corning, USA), cut into approximately 1 mm× 2 mm× 1 mm pieces and placed on the surface of T25 culture flasks (Corning, USA) moistened with DMEM medium (HyClone, USA) containing 12% fetal bovine serum (FBS) (BI, USA) and 1% penicillin/streptomycin (HyClone, USA). The flasks were inverted overnight, and on the second day, 3 ml of 12% FBS-DMEM medium was added. The cultures were maintained at 37 ℃ in a 5% CO_2_ incubator. HSFs between passages 3 and 6 were used for further experiments.

#### Cell viability and proliferation assays

HSFs were treated with ATRA at different concentrations for 24, 48, and 72 h, and cell viability was assessed using the CCK-8 assay (Beyotime, China) according to the manufacturer’s instructions. To evaluate cell proliferation, an EdU incorporation assay was performed using the EdU kit (Beyotime, China). HSFs from different treatment groups were seeded into 24-well plates and incubated with EdU reagent (1:1000 dilution) for 4 h. After staining, EdU-positive cells were visualized using an inverted fluorescence microscope and analyzed with ImageJ software.

In addition to the aforementioned materials and methods, a comprehensive description of supplementary materials and methods on source and identification of animals, histological analysis of scar tissue, HE staining, Masson’s trichrome staining, RNA isolation and quantitative real-time PCR, cell viability and proliferation assays, scratch wound healing assay, Transwell assay, collagen gel contraction assay, cell cycle analysis, apoptosis assay, and Western blotting analysis, and so on can be found in Additional file 1: Materials and methods.

### Statistical analysis

#### Statistical methods of RCT

The trial was designed to evaluate the non-inferiority of 0.05% tretinoin cream relative to silicone gel in reducing the incidence of hypertrophic scarring over a 3-month period. The sample size was determined based on a non-inferiority margin of an absolute risk difference (ARD) of 8% (Δ<8%). Sample size determinations were made assuming a one-sided α of 0.05, a power (1–β) of 0.80, a control group proportion of 40%, and an actual difference between the tretinoin group and the silicone group of –15%, and accounting for a maximum attrition rate of 10% as previously described [31]. Ultimately, a total of 112 participants were enrolled in this trial. Sample size calculations were performed using PASS 11.0 software. Continuous variables in this study, such as age, BMI, and incision length or width, are presented as mean±standard deviation (SD), while categorical variables, including gender, skin incision location, and the occurrence of side effects, are described as *n* (%).

Non-inferiority was established when the upper limit of the 90% two-sided confidence interval (CI) for the group difference in ARD was below the non-inferiority margin of Δ<8% in the intent-to-treat (ITT) analysis set. For participants with multiple incisions (2 in the silicone group and 2 in the tretinoin group), one incision from each participant was randomly selected for ITT analysis. The multiple imputation (MI) method was employed to address the missing data for the primary endpoint due to the loss to follow-up, and a sensitivity analysis was conducted by imputing the outcomes of participants who failed to complete follow-up as HS formation. Differences in the modified VSS and POSAS scores were analyzed using the Wilcoxon rank-sum test, while Chi-square and Fisher’s exact tests were used to compare the incidence of side effects between groups.

#### Statistical methods for mechanistic studies

For transcriptome sequencing, differential expression analysis was conducted using DESeq2 or DEGseq. DEGs with |log_2_ fold change (FC)|≥1 and false discovery rate (FDR)≤0.05 (DESeq2) or FDR≤0.001 (DEGseq) were considered significantly different. For metabolome sequencing, metabolites with a variable importance in projection (VIP) value>1 and *P*-value<0.05, as determined by the Student’s *t*-test, were considered significantly differentially expressed metabolites (DEMs). In addition, differences in *in vitro* imaging and histological data and *in vivo* experimental data were analyzed using the Student’s *t*-test for normally distributed data and the Wilcoxon rank-sum test for non-normally distributed data in two-group comparisons. For multiple group comparisons, ANOVA was used for normally distributed data, and the Kruskal-Wallis *H* test was employed for non-normally distributed data. Data normality was assessed using the Shapiro-Wilk test, with *P*-value>0.05 indicating normal distribution. *P*-value<0.05 was considered statistically significant. All statistical analyses were conducted using GraphPad Prism 8.0.1 software.

## Results

### Characteristics of participants included in the RCT

In this RCT, a total of 112 participants were randomly allocated to the silicone group (*n=*58) and the 0.05% tretinoin group (*n=*54). Participants were randomly recruited from three institutions: Xijing Hospital (accounting for 36.1% of the incisions), Xi’an Central Hospital (31.5%), and Second People’s Hospital of Jiangyou City (32.4%). A total of 106 participants have completed the 3-month treatment and follow-up period. The baseline demographic and clinical characteristics of the participants were summarized in Table 1. The demographic distribution and clinical features, such as the location and size of incisions, were comparable between the silicone and tretinoin groups (*P*>0.05).

**Table 1:** Demographics and baseline characteristics for the ITT set.

**Item**	**Silicone (*****n=*****58)**	**Tretinoin (*****n=*****54)**	***P-*****value**
Age (year, mean±SD)	36.28±12.23	36.52±11.96	0.915
Gender [*n* (%)]			
Male	12 (20.69)	11 (20.37)	0.967
Female	46 (79.31)	43 (79.63)	
Location of skin incisions [*n* (%)]			
Neck	12 (20.69)	11 (20.37)	0.893
Trunk	29 (50.00)	25 (46.30)	
Extremity	17 (29.31)	18 (33.33)	
BMI (kg/m^2^, mean±SD)	22.51±3.39	23.02±3.80	0.740
Size of incisions (mm, mean±SD)			
Length	55.60±55.57	48.57±38.35	0.909
Width	1.44±0.45	1.52±0.52	0.565

ITT. Intent-to-treat; BMI. Body mass index

### Evaluation of hypertrophic scarring after a 3-month treatment

**Fig. 1:**
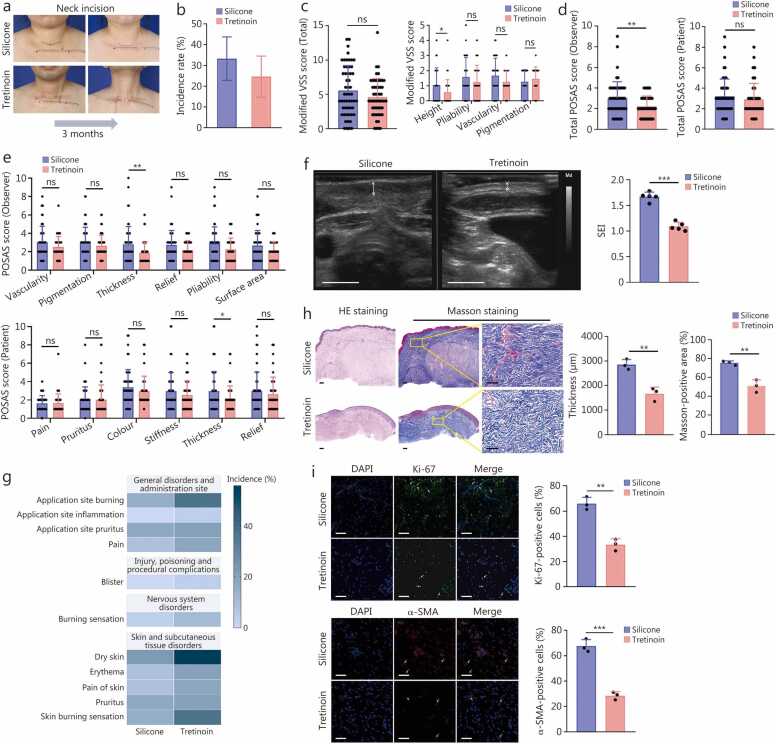
**Comparison of the efficacy of silicone gel vs. tretinoin cream in preventing postoperative hypertrophic scar formation in a multicenter RCT**. **a** Representative images of the neck incisions before and after silicone gel or 0.05% tretinoin cream treatment for 3 months, respectively. **b** Bar graph showing the incidence rates and 90% CI of HS formation in silicone gel and tretinoin cream treatment groups. **c** Comparison of total and component scores on the modified VSS between the silicone gel and tretinoin cream treatment groups. **d, e** Differences in overall and component scores as assessed by blinded observers and patients on scar appearance between the silicone and tretinoin treatment groups, utilizing the observer POSAS. **f** Representative images of the healing wounds detected by ultrasound scan after 3-month silicone gel treatment or tretinoin cream treatment, and differences in SEI measured by skin ultrasound examination between the silicone gel and tretinoin cream treatment groups (*n=*5). Scale bar = 10 mm. White arrows indicated the thicknesses of dermis. **g** Heat maps represent the incidence of incisional or peri-incisional side effects after treatment with silicone gel or tretinoin cream. **h** Representative images of HE and Masson’s trichrome staining of scar sections in the silicone gel treatment and tretinoin cream treatment groups, and the quantification of thickness and the proportion of Masson-stained areas of scar sections in the silicone gel treatment and tretinoin cream treatment groups (*n=*5). Scale bar = 200 μm. **i** IF staining of Ki-67 (top) and α-SMA (bottom) in scar sections and quantification of the proportion of Ki-67-positive and α-SMA-positive fibroblasts in the silicone gel treatment and tretinoin cream treatment groups. Scale bar = 200 μm. ^⁎⁎^*P*<0.01, ^⁎⁎⁎^*P*<0.001, ns non-significant. RCT. Randomized controlled trial; HS. Hypertrophic scar; VSS. Vancouver Scar Scale; POSAS. Patient and Observer Scar Assessment Scale; SEI. Scar elevation index; HE. Hematoxylin and eosin; IF. Immunofluorescence; α-SMA. α-smooth muscle actin.

The observers systematically assessed the appearance of healed incisions following 3 months of treatment at various high-tension surgical sites (Fig. 1**a;** Additional file 1: Fig. S2). The incidence of hypertrophic scarring in incisions treated with 0.05% tretinoin cream (24.63%) was non-inferior to that treated with the silicone gel (33.28%) (Fig. 1**b**), with an ARD of –8.65% (90% CI –23.03 to 5.74), which was below the prespecified 8% non-inferiority margin. The sensitivity analysis showed that the ARD was –10.15% (90% CI –27.43 to 7.13) (Additional file 1: Table S5**)**. Although no significant differences were observed in total VSS scores between groups, the Tretinoin group still exhibited significantly lower scores for height compared to the Silicone group (*P*<0.05; Fig. 1**c**). Besides, the POSAS scores showed that observer-assessed total scores were significantly lower in the Tretinoin group relative to the Silicone group (*P*<0.01), while no significant differences were observed in patient-assessed scores (Fig. 1**d**). Specifically, the thicknesses for POSAS scores were significantly lower in the Tretinoin group for both observer and patient assessments (*P*<0.05; Fig. 1**e**).

To objectively assess scar hyperplasia at incision sites, 5 healed incisions from each treatment group were randomly selected for skin ultrasound analysis, and a significant reduction in the SEI was observed in the Tretinoin group (Fig. 1**f**). However, compared with silicone gel, the application of 0.05% tretinoin cream was associated with a higher rate of side effects, as depicted in the heat map (Fig. 1**g**) and detailed in Additional file 1: Tables S6-S8. Notably, skin burning and dryness were more frequently reported. Most side effects in both groups were mild (66.67%–100.00%), with a small portion being moderate (0%–33.33%), while no severe side effects were observed (Additional file 1: Table S7). Moreover, all side effects associated with either treatment were self-limiting and well-tolerated (Additional file 1: Table S9). In Additional file 1: Table S8, the side effects associated with topical silicone gel typically resolved within 5 d, while those of topical tretinoin cream persisted for approximately no more than 10 d. No patients required medications to alleviate the side effects, and no withdrawals from the trial occurred due to drug-related adverse reactions. These results confirmed that 0.05% tretinoin cream could reduce HS formation, primarily by decreasing scar thickness. The observed side effects are consistent with the known profile of 0.05% tretinoin cream in the treatment of other dermatological conditions, such as psoriasis and cystic acne. These effects were typically self-limiting and well-tolerated, thus not affecting the safety and clinical applicability of the medication.

### Tretinoin cream inhibits HSF proliferation, myofibroblast activation, and collagen deposition in human HSs

To assess histological alterations in HSs, histological analysis of human scar tissues excised from patients after silicone gel or 0.05% tretinoin cream treatments revealed that 0.05% tretinoin cream significantly reduced both scar thickness [(2856.20±211.83) vs. (1664.57±273.50) μm] and collagen deposition (Fig. 1**h**). IF staining for Ki-67 and α-SMA revealed a lower proportion of Ki-67-positive and α-SMA-positive fibroblasts in tretinoin cream-treated scars, suggesting that tretinoin cream reduces HS formation by inhibiting myofibroblast activation and HSF proliferation (Fig. 1**i**).

### Tretinoin cream facilitates scarless wound healing across different phases in various wound healing models

**Fig. 2:**
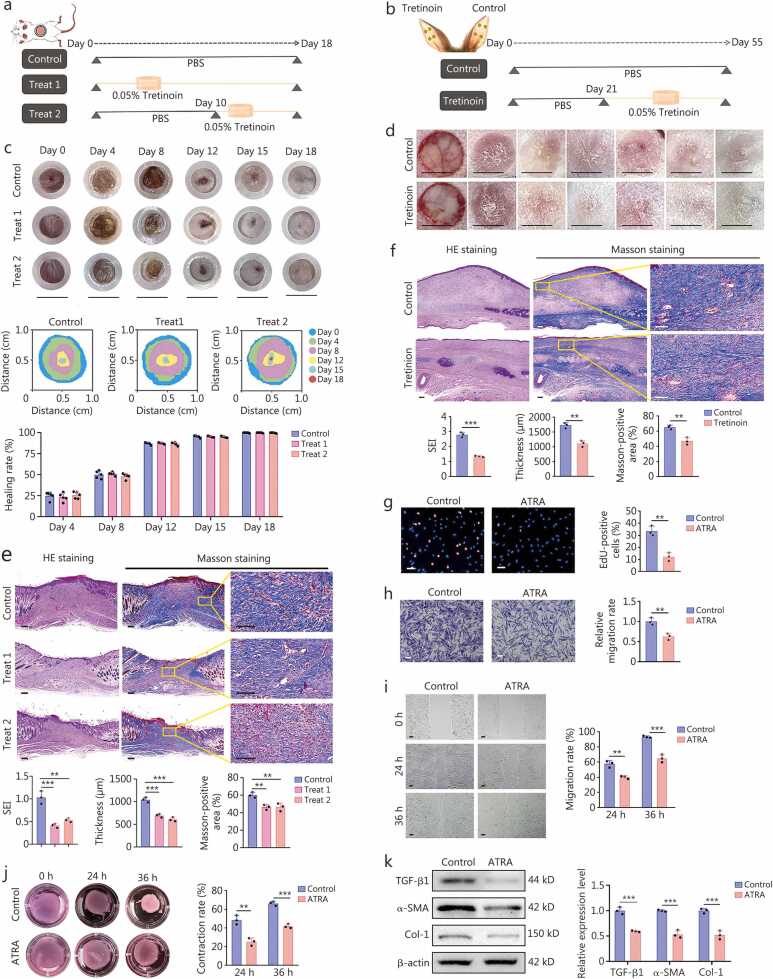
**The effects of tretinoin cream on the proliferation of fibroblasts, activation of myofibroblasts, and wound healing**. **a** A schematic overview of the mouse wound splinting model treated with PBS or 0.05% tretinoin cream. The wounds in the Control group and the Treat 1 group were treated with PBS or 0.05% tretinoin cream at individual time points, respectively, while the wounds in the Treat 2 group were treated with PBS for the first 10 d during wound healing, then the wounds were subjected to 0.05% tretinoin cream therapy (*n=*5). **b** Schematic overview of the rabbit ear model subjected to PBS or 0.05% tretinoin cream treatment. **c** Representative images of wounds, schematics illustrating the wound healing processes, and quantification of wound healing rates are presented for wounds treated with either PBS or 0.05% tretinoin cream at different time points. PBS-treated wounds served as the control group, while wounds treated with tretinoin cream starting on day 0 and day 10 post-operatively constituted the Treat 1 and Treat 2 groups, respectively. Scale bar = 8 mm. **d** Representative images of hypertrophic scar formation of rabbit ears after PBS and 0.05% tretinoin cream treatment at the indicated time points. PBS-treated and 0.05% tretinoin cream-treated wounds on day 21 post-operation served as the control group and tretinoin group, respectively. **e** Representative images of HE and Masson’s trichrome staining of mouse sections of healed wounds, along with the quantification of SEI, thickness, and proportions of Masson-positive areas of healed wounds at day 18 after wound construction in different groups. Scale bar = 200 μm. **f** Representative images of HE and Masson’s trichrome staining of rabbit ear scar sections at day 55, along with the quantification of SEI, thickness, and proportions of Masson-positive areas of scar samples after wound creation in different groups. Scale bar = 200 μm. **g** Representative images of EdU staining and quantification of the proportions of EdU-positive cells are shown for both PBS (Control group) and ATRA-treated HSFs (ATRA group) (*n=*3). Scale bar=100 μm. **h** Representative images of the Transwell assay and quantification of relative migration rates are shown for both PBS and ATRA-treated HSFs at 36 h (*n=*3). Scale bar=100 μm. **i** Representative images of scratch assay and quantification of migration rates are shown for both PBS and ATRA-treated HSFs at 24 and 36 h (*n=*3). Scale bar=100 μm. **j** Representative images of collagen gel contraction assay and quantification of relative contraction rates are shown for both PBS and ATRA-treated HSFs at 24 and 36 h (*n=*3). **k** Western blotting assay and quantification were used to detect the relative expression of TGF-β1, α-SMA, and Col-1 proteins in PBS- and ATRA-treated HSFs. ^⁎⁎^*P*<0.01, ^⁎⁎⁎^*P*<0.001. PBS. Phosphate-buffered saline; SEI. Scar elevation index; HE. Hematoxylin and eosin; ATRA. All-trans retinoic acid; HSFs. Hypertrophic scar fibroblasts; TGF-β1. Transforming growth factor-β1; α-SMA. α-smooth muscle actin; Col-1. Collagen type I.

To assess the preventative effect of 0.05% tretinoin cream on HS formation and its influence on normal cutaneous wound healing, we utilized both a mouse wound splinting model and a rabbit ear scar model. In the mouse model, tretinoin cream was applied on days 0 and 10, respectively (Fig. 2**a**), corresponding to the early and proliferative phases of wound healing, while in the rabbit ear model, treatment was initiated at day 21 post-operation, which corresponded with the tissue remodeling phase of wound healing (Fig. 2**b**). Treatment with 0.05% tretinoin cream reduced scar area by approximately 40% in mice and 45% in rabbits, with significantly lower blood perfusion (Additional file 1: Fig. S3a-d). Notably, the treatment did not affect the appearance of healed wounds or cause delayed wound healing (Fig. 2**c, d**). Histological examination revealed reduced scar thickness and collagen deposition, accompanied by lower SEI in both models (Fig. 2**e, f**). Besides, IF staining indicated a lower proportion of α-SMA-positive myofibroblasts in treated scars (Additional file 1: Fig. S3e).

### ATRA suppresses the proliferation and myofibroblast transformation in HSFs

ATRA, the primary active ingredient in tretinoin cream, has been utilized in the treatment of various medical conditions [32], [33], and ATRA inhibited the proliferation of HSFs in a concentration-dependent manner. To determine the optimal inhibitory concentration, a CCK-8 assay was performed. Specifically, nonlinear regression fitting was applied to calculate the half-maximal inhibitory concentration (IC_50_) based on the relative cell viability of HSFs treated with varying concentrations of ATRA for 72 h, compared with that of the untreated control group. However, the fitting efficacy was suboptimal, yielding a derived IC_50_ value of 40.17 μg/ml (Additional file 1: Fig. S4a). Concurrently, by integrating relative cell viability data across multiple time points, we found that only the concentration of 50 μg/ml, exceeding the predicted IC_50_ value, exerted a significant inhibitory effect on HSF proliferation at all time points tested (Additional file 1: Fig. S4b). Thus, the optimal inhibitory concentration of ATRA was ultimately determined to be 50 μg/ml. At this concentration, ATRA suppressed HSF proliferation, lateral and longitudinal migration, and collagen gel contraction (Fig. 2**g-j**). Besides, ATRA increased the proportion of apoptotic cells and induced a G1 cell-cycle arrest, leading to a subsequent decrease in the S phase (Additional file 1: Fig. S4c, d). Moreover, ATRA decreased the expression of TGF-β1, α-SMA, and collagen type I (Col-1) (Fig. 2**k;** Additional file 1: S4e), further supporting its role in inhibiting myofibroblast transformation and collagen synthesis of HSFs.

### Integrative analysis of transcriptome and metabolome in HSFs after ATRA treatment

To investigate the underlying mechanisms by which ATRA inhibits the activation and proliferation of HSFs, we performed transcriptomic and metabolomic analysis on HSFs treated with 50 μg/ml ATRA or PBS (Additional file 1: Table S4). Principal component analysis (PCA) revealed distinct clustering of ATRA-treated samples (Additional file 1: Fig. S5a). A total of 5171 DEGs (upregulated: 2878 genes; downregulated: 2293 genes) and 232 DEMs (upregulated: 72 metabolites; downregulated: 160 metabolites) were identified (Additional file 1: Fig. S5b). KEGG enrichment revealed that the DEGs and DEMs were enriched in key pathways related to glycolysis/gluconeogenesis, OXPHOS, glutathione metabolism, and pathways related to the TCA cycle (Additional file 1: Fig. S5c).

### Glycolysis reprogramming in myofibroblast activation and proliferation in HSFs

**Fig. 3:**
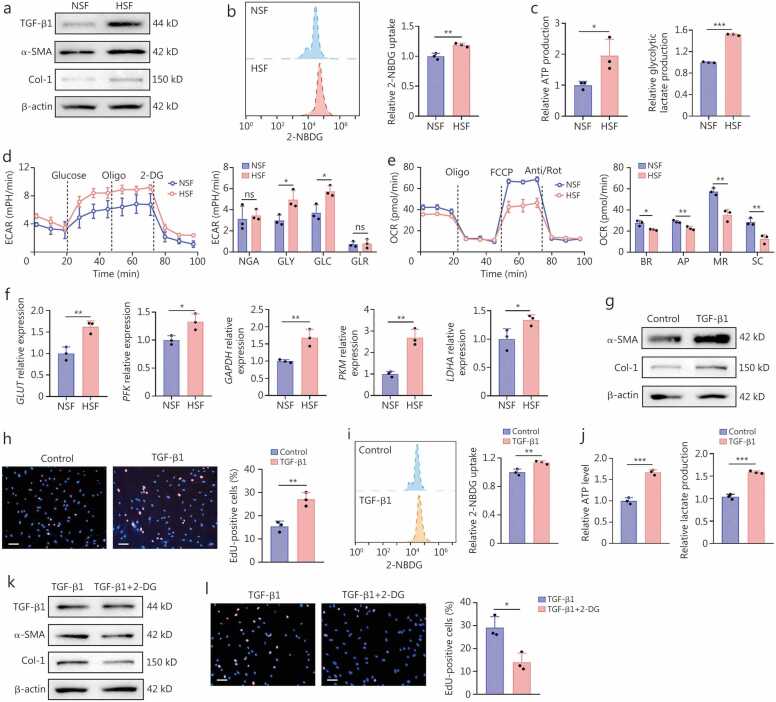
**The relationship between myofibroblast activation and glycolysis reprogramming**. **a** Western blotting assay was used to detect TGF-β1, α-SMA, and Col-1 protein expression of the fibroblasts isolated from normal skin (NSF) and hypertrophic scar (HSF). **b** Flow cytometry analysis and quantification of fluorescent 2-NBDG uptake in NSF and HSF. **c** Differences in ATP and glycolytic lactate production capacities between the NSF and HSF. **d** Extracellular acidification rate (ECAR) of NSF and HSF, and quantification of non-glycolytic acidification (NGA), glycolysis (GLY), glycolytic capacity (GLC), and glycolytic reserve (GLR). **e** Mitochondrial oxygen consumption rate (OCR) of NSF and HSF, and quantification of basal OCR (BR), ATP-linked OCR (AP), maximal OCR (MR), and spare OCR (SC). **f** Differences in the mRNA expressions of glycolytic enzymes between NSF and HSF were detected by qRT-qPCR. **g** Western blotting assay was applied to detect α-SMA and Col-1 protein expression of the NSF with and without TGF-β1 stimulation. **h** Representative images of EdU staining and quantification of the proportions of EdU-positive cells are shown for both PBS (Control group) and TGF-β1-stimulated NSFs (TGF-β1 group) (*n=*3). Scale bar=100 μm. **i** Flow cytometry analysis and quantification of fluorescent 2-NBDG uptake are shown for both PBS and TGF-β1-stimulated NSFs. **j** Differences in ATP production capacities and glycolytic lactate production between PBS and TGF-β1-stimulated NSF. **k** Western blotting assay was harnessed to detect α-SMA and Col-1 protein expression of the NSF exposed to 10 ng/ml TGF-β1 with or without 10 mmol/L 2-DG treatment. **l** Representative images of EdU staining and quantification of the proportions of EdU-positive NSFs stimulated with TGF-β1 with or without 2-DG (*n=*3). Scale bar=100 μm. ^⁎^*P*<0.05, ^⁎⁎^*P*<0.01, ^⁎⁎⁎^*P*<0.001. TGF-β1. Transforming growth factor-β1; α-SMA. α-smooth muscle actin; Col-1. Collagen type I; RT-qPCR. Quantitative reverse transcription PCR; 2-DG. 2-deoxy-D-glucose; 2-NBDG. 2-deoxy-2- [(7-nitro-2,1,3-benzoxadiazol-4-yamino]-D-glucose.

Integrative omics analysis revealed that ATRA could significantly inhibit myofibroblast activation by modulating glucose and energy metabolism. However, the relationships between abnormal metabolic reprogramming and both proliferation and myofibroblast activation of HSFs warrant further investigation. HSFs exhibited elevated levels of TGF-β1, α-SMA, and Col-1 proteins compared to NSFs (Fig. 3**a;** Additional file 1: Fig. S6a). Elevated expressions of TGF-β1, α-SMA, and Col-1 in fibroblasts are indicative of myofibroblast proliferation and activation, which play a significant role in the pathogenesis of HS. Notably, compared to the NSFs, the levels of 2-NBDG uptake, ATP production, and intracellular lactate production were increased in HSFs (Fig. 3**b, c**). Besides, Seahorse assays revealed higher glycolytic activity and reduced mitochondrial respiration in HSFs, as indicated by elevated ECAR and decreased OCR (Fig. 3**d, e**). Consistently, RT-qPCR revealed an upregulation of key glycolytic enzymes, including *GLUT*, *PFK*, *GAPDH*, *PKM*, and *LDHA* in HSFs (Fig. 3**f**). These results suggest a metabolic shift from mitochondrial respiration to glycolysis during the transition from NSFs to HSFs.

To elucidate the potential interactions between glycolysis reprogramming and myofibroblast activation, we assessed the metabolic characteristics of normal skin fibroblasts (NSFs) upon TGF-β1 treatment, which stimulated myofibroblast activation and proliferation (Fig. 3**g, h;** Additional file 1: Fig. S6b). Simultaneously, TGF-β1 enhanced NSF 2-NBDG uptake, ATP production, and lactate production (Fig. 3**i, j**), indicating that TGF-β1-stimulated myofibroblast activation promoted glycolysis. Furthermore, 2-DG, a specific inhibitor of glycolysis, was used to treat NSFs following TGF-β1 stimulation, which led to a decrease in α-SMA and Col-1 expression and reduced cell proliferation in NSFs (Fig. 3**k, l; A**Additional file 1: Fig. S6c), while TGF-β1 levels remained unchanged. These results suggested that glycolysis is a key process in TGF-β1-induced myofibroblast activation, and inhibiting glycolysis can suppress fibroblast-to-myofibroblast (FTM) transition and fibroblast proliferation.

### ATRA alleviates glucose metabolism dysregulation in HSFs

**Fig. 4:**
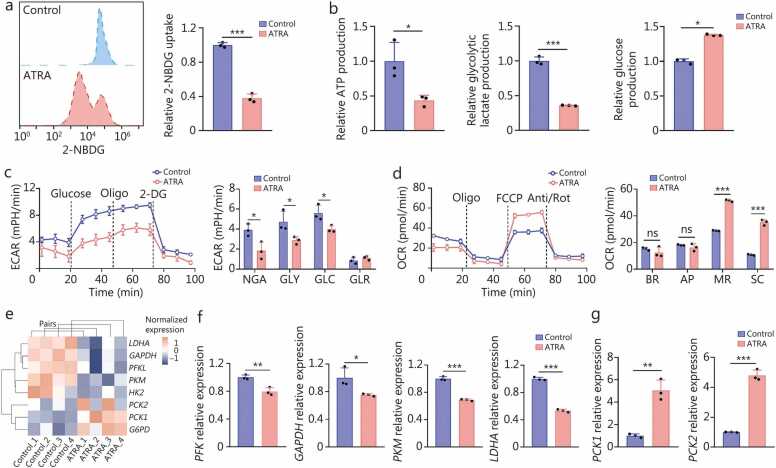
**All-trans retinoic acid (ATRA) promotes gluconeogenesis while inhibiting glycolysis in fibroblasts isolated from hypertrophic scar samples**. **a** Flow cytometry analysis and quantification of fluorescent 2-NBDG uptake are shown for both PBS- (Control group) and ATRA-treated (ATRA group) HSFs (*n=*3). **b** Differences in ATP production capacities, glycolytic lactate production, and glucose production capacities between PBS- and ATRA-treated HSFs. **c** ECAR of PBS- and ATRA-treated HSFs, and quantification of their NGA, GLY, GLC, and GLR. **d** OCR of PBS and ATRA-treated HSFs, and quantification of their BR, AP, MR, and SC. **e** A heat map represents the differential expression of key enzymes in glucose metabolism for PBS- and ATRA-treated HSFs. **f, g** Differences in the mRNA expression of glycolytic (**f**) and gluconeogenic (**g**) enzymes between PBS- and ATRA-treated HSFs via RT-qPCR (*n=*3). ^⁎^*P*<0.05, ^⁎⁎^*P*<0.01, ^⁎⁎⁎^*P*<0.001. PBS. Phosphate-buffered saline; ATP. Adenosine triphosphate; ECAR. Extracellular acidification rate; NGA. Non-glycolytic acidification; GLY. Glycolysis GLC. Glycolytic capacity; GLR. Glycolytic reserve; OCR. Oxygen consumption rate; BR. Basal OCR; AP. ATP-linked OCR; MR. Maximal OCR; SC. Spare OCR; RT-qPCR. Quantitative reverse transcription PCR; HSFs. Hypertrophic scar fibroblasts.

Based on multi-omics analysis, further validations on glucose metabolism confirmed that ATRA decreased 2-NBDG uptake, ATP production, and intracellular lactate production, while enhancing glucose production in HSFs (Fig. 4**a, b**). Seahorse analyses revealed that ATRA increased mitochondrial respiration and inhibited glycolysis (Fig. 4**c, d**). These findings suggested that ATRA reprograms glucose metabolism by suppressing glycolysis and promoting gluconeogenesis, thereby restoring OXPHOS as the primary energy source in HSFs. Furthermore, following the ATRA treatment, the heatmap revealed decreased expression of key glycolytic enzymes (including *PFKL*, *GAPDH*, *PKM*, and *LDHA*), accompanied by the increased expression of key gluconeogenic enzymes (*PCK1*, *PCK2*, *G6PD*) (Fig. 4**e**). Meanwhile, RT-qPCR also confirmed the downregulation of key glycolytic enzymes (*PFK*, *GAPDH*, *PKM,* and *LDHA*) and upregulation of key gluconeogenic enzymes (*PCK1*, *PCK2*) in ATRA-treated HSFs (Fig. 4**f, g**). By detecting glycolysis levels and potential key enzymes involved in glucose metabolism, we confirmed that ATRA modulates the proliferation and activation of HSFs through glucose metabolism remodeling. Moreover, identifying upstream core molecules that regulate glucose metabolic processes and multiple key enzymes, as well as elaborating on their regulatory patterns, is of great significance for clarifying the mechanism underlying ATRA-mediated inhibition of hypertrophic scarring.

### ATRA inhibits the activation and proliferation and reduces glycolysis of HSFs by upregulating HIC1 expression

**Fig. 5:**
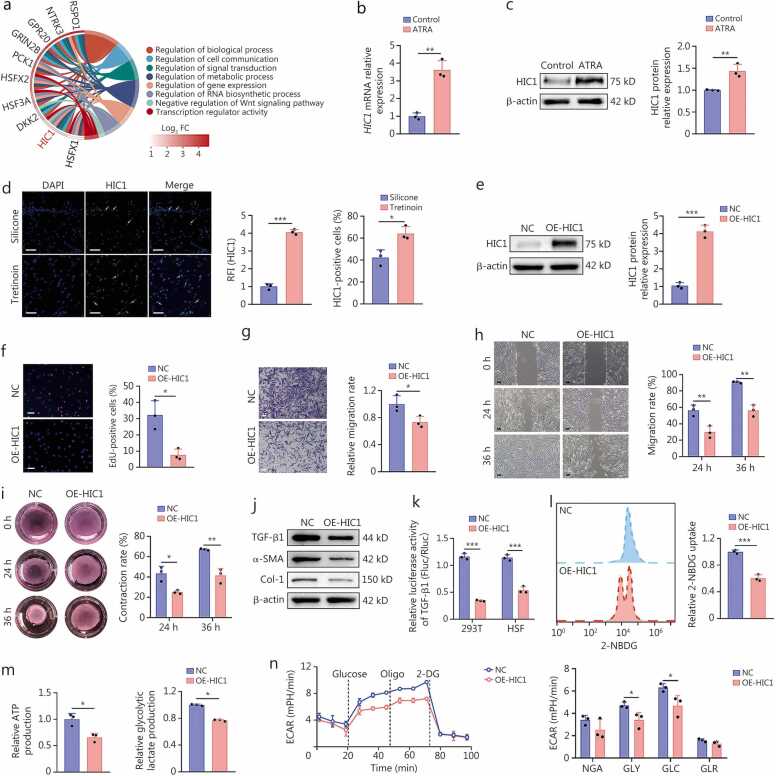
**The upregulation of HIC1 in HSFs inhibits glycolysis and the transition of HSFs to myofibroblasts**. **a** Chord plot showing the enriched GO pathways and involved DEGs in ATRA-treated HSFs. **b** Differences in HIC1 expression between PBS (Control group) and ATRA-treated (ATRA group) HSFs detected by RT-qPCR (*n=*3). **c** Western blotting assay and quantification of HIC1 protein levels of PBS (Control group) and ATRA-treated (ATRA group) HSFs (*n=*3). **d** IF staining of HIC1 protein in human hypertrophic scar tissue sections and quantification of the relative fluorescence intensity (RFI) of HIC1 and proportion of HIC1-positive HSFs in the silicone gel treatment and tretinoin cream treatment groups (*n=*3). Scale bar = 200 μm. White arrows indicated the representative images of HIC1-positive cells. e Western blotting assay and the quantification of HIC1 protein expression in HSFs treated with either a negative control vector (NC group) or an HIC1 overexpression vector (OE-HIC1 group) via transfection. **f** Representative images of EdU staining and quantification of the proportions of EdU-positive cells (*n=*3). Scale bar=100 μm. **g** Representative images of the transwell assay and quantification of relative migration rates of HSFs (*n=*3). Scale bar=100 μm. **h** Representative images of scratch assay and quantification of migration rates of HSFs at 24 and 36 h (*n=*3). Scale bar=100 μm. **i** Representative images of collagen gel contraction assay and quantification of relative contraction rates of HSFs at 24 and 36 h (*n=*3). **j** Western blotting assay was used to detect TGF-β1, α-SMA, and Col-1 protein expression in HSFs after transfection with a control vector and *HIC1* overexpression vector. **k** Luciferase reporter assays were performed to evaluate TGF-β1 transcriptional activity in 293 T cells and HSFs. These cells were transfected with a pcDNA3.1 (pc3.1) plasmid encoding either a control vector or HIC1, in conjunction with *TGF-β1* promoter vectors. **l** Flow cytometry analysis and quantification of fluorescent 2-NBDG uptake in HSFs are presented for both the negative control and HIC1 overexpression groups. **m** Differences in ATP production and glycolytic lactate production capacities in HSFs for both the negative control and HIC1 overexpression groups (*n=*3). **n** ECAR of HSFs in both the negative control and HIC1 overexpression groups, and quantification of their NGA, GLY, GLC, and GLR (*n=*3). ^⁎^*P*<0.05, ^⁎⁎^*P*<0.01, ^⁎⁎⁎^*P*<0.001. HSFs. Hypertrophic scar fibroblasts; HIC1. Hypermethylated in cancer 1; GO. Gene Ontology; DEGs. Differentially expressed genes; ATRA. All-trans retinoic acid; PBS. Phosphate-buffered saline; IF. Immunofluorescence; OE. Overexpression; RT-qPCR. Quantitative reverse transcription PCR; TGF-β1. Transforming growth factor-β1; α-SMA. α-smooth muscle actin; Col-1. Collagen type I; ATP. Adenosine triphosphate; ECAR. Extracellular acidification rate; NGA. Non-glycolytic acidification; GLY. Glycolysis; GLC. Glycolytic capacity; GLR. Glycolytic reserve.

Previous studies have confirmed that cellular communication, signaling, and metabolism are crucial for HS formation [34], [35]. In the present study, we found that HIC1 was implicated in regulating these processes in HSFs (Fig. 5**a**). Treatment with tretinoin cream or its primary bioactive component (ATRA), resulted in elevated *HIC1* mRNA and protein expression both *in vitro* and *in vivo* compared with the control groups (treated with silicone gel or PBS), with IF staining confirming its nuclear localization in human and mouse scar tissues (Fig. 5**b-d;** Additional file 1: Fig. S7a-c). To examine the effects of HIC1 upregulation on HSFs, we overexpressed the *HIC1* gene in HSFs (Fig. 5**e**). HIC1 overexpression significantly inhibited cell proliferation, migration, and collagen gel contraction (Fig. 5**f-i)**, accompanied by early apoptosis and cell cycle arrest in the G0/G1 phase (Additional file 1: Fig. S7d, e). Besides, *HIC1* overexpression reduced the expression of TGF-β1, α-SMA, and Col-1 proteins (Fig. 5**j,** Additional file 1: Fig. S8a). Furthermore, the binding motif for the transcriptional repressor HIC1 and its predicted top 3 binding sites within the TGF-β1 promoter (-2000 to -1873 bp, -1307 to -1044 bp, -901 to -696 bp) were predicted using the JASPAR database (http://jaspardev.genereg.net/). These predictions were validated through chromatin immunoprecipitation followed by quantitative PCR (ChIP-qPCR) (Additional file 1: Fig. S8b, c), and the luciferase reporter assay showed that HIC1 significantly suppressed the transcriptional activity of TGF-β1 (Fig. 5**k**; Additional file 1: Fig. S8d). Besides, overexpression of *HIC1* in HSFs reduced 2-NBDG uptake, ATP production, and lactate production, along with a decline in ECAR, collectively (Fig. 5**l-n**). Moreover, HIC1 downregulated the key glycolytic enzymes such as *HK2*, *GAPDH*, and *LDHA* (Additional file 1: Fig. S8e). These findings collectively demonstrated that HIC1 could attenuate myofibroblast activation and HSF proliferation by inhibiting TGF-β1 expression and glycolysis.

### ATRA promotes gluconeogenesis and inhibits HSF activation and proliferation by upregulating PCK1 and PCK2 expression

**Fig. 6:**
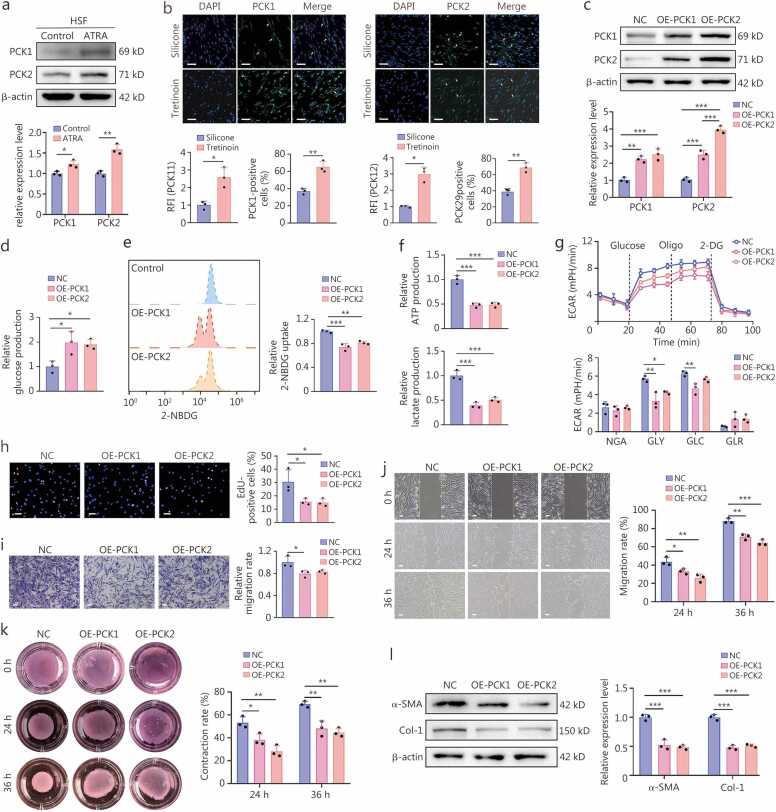
**The upregulation of PCK1 and PCK2 in HSFs reprogrammed the glycolysis/gluconeogenesis process and hampered HSF activation**. **a** Western blotting assay and quantification of PCK1 and PCK2 protein levels in HSFs with PBS (Control group) or ATRA (ATRA group) treatment. **b** Immunofluorescence staining for PCK1 and PCK2 proteins in human scar sections, and quantification of their relative fluorescence intensity (RFI) and the proportion of PCK1/PCK2-positive fibroblasts, were conducted in the Silicone and Tretinoin groups (*n=*3). Scale bar = 200 μm. White arrows indicated the representative images of PCK1-positive and PCK2- positive cells. **c** Western blotting assay and the quantification of PCK1 and PCK2 protein expression in HSFs treated with a negative control vector (NC group), a *PCK1* overexpression vector (OE-PCK1 group), and a *PCK2* overexpression vector (OE-PCK2 group), respectively (*n=*3). **d** Differences in glucose production capacities in HSFs are presented for the NC, OE-PCK1, and OE-PCK2 groups (*n=*3). **e** Flow cytometry analysis and quantification of fluorescent 2-NBDG uptake in HSFs are presented for the NC, OE-PCK1, and OE-PCK2 groups (*n=*3). **f** Differences in ATP production capacities and glycolytic lactate production in HSFs are presented for the NC, OE-PCK1, and OE-PCK2 groups (*n=*3). **g** ECAR of HSFs in NC, OE-PCK1, and OE-PCK2 groups, and quantification of their NGA, GLY, GLC, and GLR (*n=*3). **h** Representative images of EdU staining and quantification of the proportions of EdU-positive HSFs are presented for the NC, OE-PCK1, and OE-PCK2 groups (*n=*3). Scale bar=100 μm. **i** Representative images of the Transwell assay and quantification of relative migration rates of HSFs are presented for the NC, OE-PCK1, and OE-PCK2 groups (*n=*3). Scale bar=100 μm. **j** Representative images of scratch assay and quantification of migration rates of HSFs at 24 and 36 h are presented for the NC, OE-PCK1, and OE-PCK2 groups (*n=*3). Scale bar=100 μm. **k** Representative images of collagen gel contraction assay and quantification of relative contraction rates of HSFs at 24 h and 36 h are presented for the NC, OE-PCK1, and OE-PCK2 groups (*n=*3). **l** Western blotting assay and quantification of α-SMA and Col-1 protein levels in HSFs transfected with a negative control vector, a *PCK1* overexpression vector, or a *PCK2* overexpression vector (*n=*3). ^⁎^*P*<0.05, ^⁎⁎^*P*<0.01, ^⁎⁎⁎^*P*<0.001. PCK1. Phosphoenolpyruvate carboxykinase 1; PCK2. Phosphoenolpyruvate carboxykinase 2; NGA. Non-glycolytic acidification; GLY. Glycolysis; GLC. Glycolytic capacity; GLR. Glycolytic reserve; ATRA. All-trans retinoic acid; α-SMA. α-smooth muscle actin; Col-1. Collagen type I; ECAR. Extracellular acidification rate 2-NBDG. 2-deoxy-2-[(7-nitro-2,1,3-benzoxadiazol-4-yamino]-D-glucose.

Intracellular glucose homeostasis is regulated by both glycolysis and gluconeogenesis [36]. Our study demonstrated that the expression of key gluconeogenic enzymes, PCK1 and PCK2, was enhanced in HSFs following ATRA treatment *in vitro* (Fig. 6**a**). *In vivo* IF staining confirmed the high expression of PCK1 and PCK2 in both human scar sections and mouse scar tissues with predominant cytoplasmic localization (Fig. 6**b;** Additional file 1: Fig. S9). To assess the impact of PCK1/PCK2 on glucose metabolism, we overexpressed these enzymes in HSFs, respectively (Fig. 6**c**). Overexpression of either PCK1 or PCK2 enhanced the expression of *PCK2* or *PCK1*, thereby synergistically promoting glucose production and suppressing reduced glucose uptake, ATP production, and lactate production, along with a decline in ECAR (Fig. 6**d-g**). Furthermore, *PCK1/PCK2* overexpression further upregulated the expression of gluconeogenic enzyme *G6PD* while downregulating the expression of key glycolytic enzymes, including *GLUT*, *HK2*, *PFK*, *PKM*, and *LDHA* (Additional file 1: Fig. S10). Overall, these findings suggest that ATRA could facilitate gluconeogenesis by upregulating PCK1 and PCK2, which in turn inhibit aerobic glycolysis. Furthermore, overexpression of either *PCK1* or *PCK2* could suppress the proliferation, migration, and collagen gel contraction of HSFs (Fig. 6**h-k**). Besides, OE-PCK1 or OE-PCK2 inhibited myofibroblast activation and collagen synthesis by downregulating α-SMA and Col-1 (Fig. 6**l**).

### ATRA activates the transcription of HIC1, PCK1, and PCK2 via RARα

**Fig. 7:**
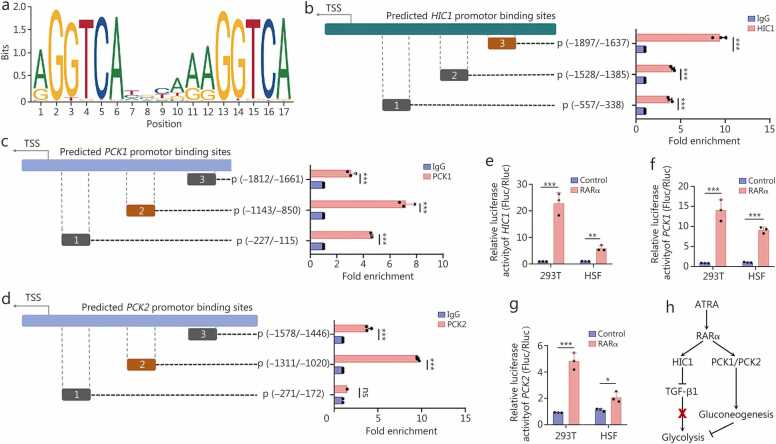
**ATRA upregulated HIC1, PCK1, and PCK2 expression through the activation of RARα in HSFs**. **a** The predicted RARα transcription factor binding motif obtained from JASPAR. **b**-**d** ChIP-qPCR assays were conducted to confirm the predicted binding sites of RARα transcription factors on the promoters of *HIC1* (**b**), *PCK1* (**c**), and *PCK2* (**d**) genes in HSFs, and the most significantly enriched regions of *HIC1*, *PCK1*, and *PCK2* promoters were coloured orange for constructing the plasmid. **e**-**g** Luciferase reporter assays were performed to evaluate *HIC1* (**e**), *PCK1* (**f**), and *PCK2* (**g**) transcriptional activity in 293 T cells and HSFs. These cells were transfected with a pcDNA3.1 (pc3.1) plasmid encoding either a control vector or RARα, in conjunction with *HIC1*, *PCK1*, and *PCK2* promoter plasmids, respectively (*n*=3). **h** Flowchart of ATRA regulating the glycolysis and gluconeogenesis pathways in the RARα-dependent manner in HSFs. ^⁎^*P*<0.05, ^⁎⁎^*P*<0.01, ^⁎⁎⁎^*P*<0.001. ATRA. All-trans retinoic acid; PCK1. Phosphoenolpyruvate carboxykinase 1; PCK2. Phosphoenolpyruvate carboxykinase 2; HIC1. Hypermethylated in cancer 1; HSFs. Hypertrophic scar fibroblasts; RARα. Retinoic acid receptor α.

ATRA typically exerts its biological effects through RARs, including RARα, RARβ, and RARγ [37]. However, the specific RARs predominantly responsible for ATRA’s regulation of the target genes *HIC1*, *PCK1*, and *PCK2* remain unclear. In this study, we utilized selective agonists (AM580, BMS641, and Palovarotene) and shRNAs to specifically activate RARα, RARβ, and RARγ [38], [39], [40] and to knock down their expression, respectively (Additional file 1: Fig. S11a-f). Two sets of shRNA sequences were designed (Additional file 1: Table S10). Activation of RARα in HSFs substantially increased the expression of all three target genes, while activation of RARβ and RARγ only moderately increased the expression of *HIC1* or *PCK2* with lower fold-changes compared to RARα (Additional file 1: Fig. S11b, d, f). Then, the JASPAR database was utilized to predict the binding motifs of RARs, as well as to assess the binding scores between RARs and their potential target genes, *HIC1*, *PCK1*, and *PCK2*, and the binding motifs for RARβ and RARγ were exclusively identified in the promoters of *HIC1* and *PCK2* (Fig. 7**a**, Additional file 1: S11g, h). Furthermore, to elucidate the RARα-dependent regulation patterns, we performed ChIP-qPCR assays and confirmed the binding of RARα to the promoters of *HIC1*, *PCK1*, and *PCK2*, which harbor multiple predicted binding sites (Fig. 7**b-d**). Afterwards, we constructed plasmids containing the most significantly enriched predicted promoter-binding sites (highlighted in yellow), namely the *HIC1* promoter region (–1897 to –1637 bp), *PCK1* promoter region (–1143 to –850 bp), and *PCK2* promoter region (–1311 to –1020 bp) (Fig. 7**b-d**). A dual-luciferase reporter assay performed using these plasmids demonstrated that transfection with a *RARα* plasmid enhanced the transcriptional activity of the specific promoter regions of *HIC1*, *PCK1*, and *PCK2* in both 293 T cells and HSFs (Fig. 7**e-g;** Additional file 1: Fig. S12). Collectively, these findings indicate that ATRA activates RARα in HSFs, which subsequently binds to the promoter regions of *HIC1*, *PCK1*, and *PCK2*, promoting their transcription (Fig. 7**h)**.

### Knockdown of *HIC1*, *PCK1*, or *PCK2* compromises the inhibitory effect of ATRA on the proliferation and activation of HSFs

To validate the inhibitory effect of ATRA on the proliferation and activation of HSFs via regulating the functional molecules comprising HIC1, PCK1, and PCK2, a series of rescue experiments was conducted. After confirming the knockdown efficiency of shRNAs targeting *HIC1*, *PCK1*, and *PCK2* (Additional file 1: Fig. S13a-c), HSFs treated with ATRA (50 μg/ml) were set as the control. Subsequently, following ATRA treatment, HSFs were further treated with shHIC1, shPCK1, and shPCK2, respectively, and cell proliferation and the expression levels of fibrosis-related proteins, including α-SMA, Col-1, and Col-3, were assessed. As illustrated in Additional file 1: Fig. S13d-f, a significant reduction in the protein expression of α-SMA, Col-1, and Col-3 was observed in HSFs following ATRA treatment, while subsequent knockdown of the functional molecules of *HIC1*, *PCK1*, or *PCK2* induced recovery of the expression levels of these fibrosis-related proteins to some extent. Similarly, EdU assays revealed that ATRA treatment significantly inhibited the proliferation of HSFs, while this inhibitory effect was remarkably reversed by the knockdown of *HIC1*, *PCK1*, or *PCK2* (Additional file 1: Fig. S13g-i**)**. Overall, these findings suggest that the upregulation of HIC1, PCK1, and PCK2 following ATRA treatment plays a crucial role in mediating ATRA’s inhibitory effects on fibroblast proliferation and activation. Furthermore, these molecules represent promising novel therapeutic targets for treating skin fibrotic diseases.

### Fibroblast-specific overexpression of *HIC1*, *PCK1* or *PCK2* reduce mice scar formation

**Fig. 8:**
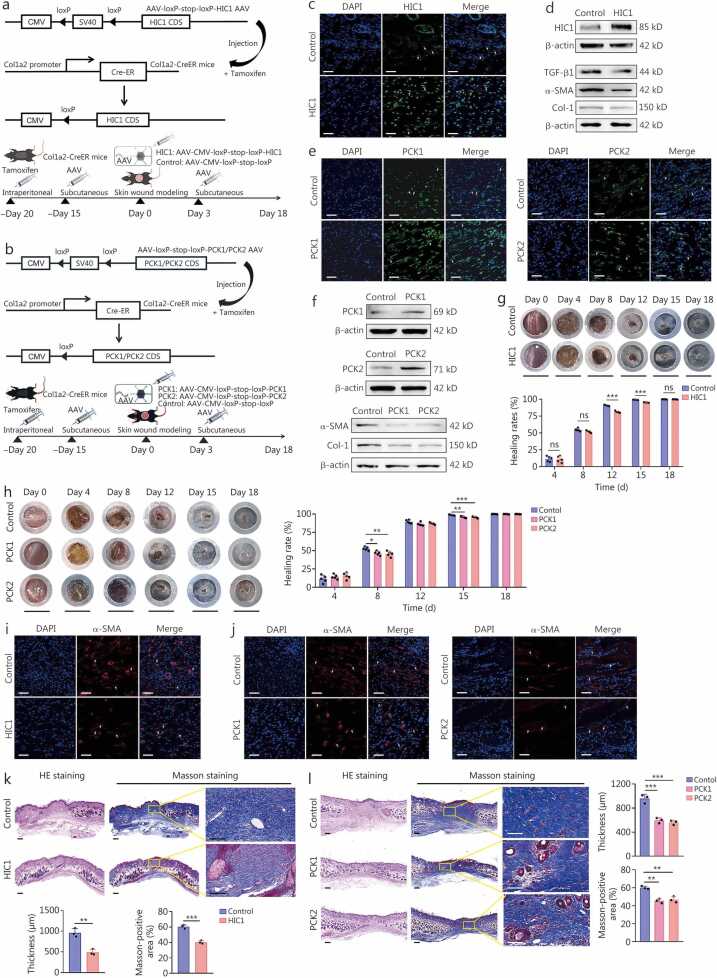
**Elevated expression of HIC1, PCK1, and PCK2 in fibroblasts mitigates scar formation in Col1a2-CreER mice, as demonstrated using the Col1a2-CreER system**. **a** A schematic illustrates the implementation of fibroblast-specific HIC1 overexpression in skin fibroblasts utilizing the Cre-loxP system, alongside the experimental timeline of the mouse wound splinting model. **b** A schematic illustrates the implementation of fibroblast-specific *PCK1* and *PCK2* overexpression in skin fibroblasts utilizing the Cre-loxP system, alongside the experimental timeline of the mouse wound splinting model. **c** Immunofluorescence staining of the HIC1 protein confirms the successful delivery of AAV-HIC1 into skin fibroblasts. This is evidenced by the elevated expression of HIC1 in fibroblasts derived from healed wounds within the AAV-HIC1 group (HIC1 group) compared with the AAV-control group (Control group) in Col1a2-CreER mice. Scale bar = 200 μm. White arrows indicate the representative HIC1-positive cells.**d** Western blotting assay was used to detect HIC1, TGF-β1, α-SMA, and Col-1 protein expression in fibroblasts isolated from the regenerated skin from the healed wounds of mice in the control group and HIC1 group. **e** IF staining of the PCK1 and PCK2 proteins confirms the successful delivery of AAV-PCK1 and AAV-PCK2 into skin fibroblasts. This is evidenced by the elevated expression of PCK1 and PCK2 in fibroblasts derived from healed wounds within the AAV-PCK1 group (PCK1 group) and AAV-PCK2 (PCK2 group) compared with the AAV-control group (Control group) in Col1a2-CreER mice. Scale bar = 200 μm. White arrows indicated the representative images of PCK1-positive and PCK2-positive cells. **f** Western blotting assay was used to detect PCK1, PCK2, α-SMA, and Col-1 protein expression in fibroblasts isolated from the regenerated skin from the original wound sites of mice in the control, PCK1, and PCK2 groups. **g** Representative images of wounds and quantification of wound healing rates are presented for wounds in the control group and HIC1 group at different time points (*n*=5). Scale bar = 8 mm. **h** Representative images of wounds and quantification of wound healing rates are presented for the control group, PCK1 group, and PCK2 group at various time points (*n*=5). Scale bar = 8 mm. **i** IF staining of α-SMA for the myofibroblasts in the sections from healed wounds of Col1a2-CreER mice in the control group and HIC1 group. Scale bar = 200 μm. White arrows indicated the representative images of α-SMA positive cells. **j** IF staining of α-SMA for the myofibroblasts in the sections from healed wounds of Col1a2-CreER mice in the control group, PCK1 group, and PCK2 group. White arrows indicated the representative images of α-SMA-positive cells. **k, l** Representative images of HE and Masson’s trichrome staining, and the quantification of thickness and the proportion of Masson-positive areas of the sections from healed wounds in the control and HIC1 groups (**k**), as well as control, PCK1, and PCK2 groups (**l**) in Col1a2-CreER mice (*n=*3). Scale bar = 200 μm. ^⁎^*P*<0.05; ^⁎⁎^*P*<0.01; ^⁎⁎⁎^*P*<0.001. HIC1. Hypermethylated in cancer 1; PCK1. Phosphoenolpyruvate carboxykinase 1; PCK2. Phosphoenolpyruvate carboxykinase 2; AAV-HIC1. AAV-CMV-loxP-stop-loxP-HIC; AAV-PCK1. AAV-CMV-loxP-stop-loxP-PCK1; AAV-PCK2. AAV-CMV-loxP-stop-loxP-PCK2; AAV-control. AAV-CMV-loxP-stop-loxP; HSFs. Hypertrophic scar fibroblasts; α-SMA. α-smooth muscle actin; Col-1. Collagen type I; IF. Immunofluorescence; HE. Hematoxylin and eosin; CMV. Cytomegalovirus; CDS. Coding DNA sequence.

To investigate the effects of fibroblast-specific overexpression of *HIC1*, *PCK1*, or *PCK2* on scar formation during wound healing *in vivo*, tamoxifen-inducible Col1a2-CreER mice were injected with AAVs carrying either *HIC1*, *PCK1*, *PCK2*, or control sequences into their dorsal skin (Fig. 8**a, b**). First, PCR analysis confirmed the presence of the Col1a2-CreER allele in mice, yielding a 186 bp product for Col1a2-CreER and a 324 bp product for the wild-type (Additional file 1: Fig. S14a). Then, IF staining and Western blotting analysis of scar tissues confirmed the fibroblast-specific overexpression of *HIC1*, *PCK1* or *PCK2* in mice based on the Cre-loxP system (Fig. 8**c-f;** Additional file 1: Fig. S14b-i). In the full-thickness skin wound model, although the injections of AAV-HIC1, AAV-PCK1, and AAV-PCK2 exhibited a slight delay in wound healing compared to the AAV-control group, complete wound healing was achieved in all groups by day 18 post-operation (Fig. 8**g, h;** Additional file 1: S15a, b). Notably, the scar areas in the HIC1, PCK1, and PCK2 groups were significantly reduced at 18 d after wound creation (Additional file 1: Fig. S15c, d). Moreover, the fibroblast-specific overexpression of these genes significantly decreased scar blood perfusion (Additional file 1: Fig. S15e, f).

### HIC1, PCK1, or PCK2 inhibits myofibroblast activation and collagen synthesis

*In vitro* experiments confirmed that *HIC1* overexpression led to a significant reduction of TGF-β1, α-SMA, and Col-1 proteins (Fig. 8**d;** Additional file 1: S14d). Similarly, overexpression of *PCK1* or *PCK2* resulted in a decrease in α-SMA and Col-1 protein (Fig. 8**f;** Additional file 1: S14h, i). Moreover, fibroblast-specific overexpression of these genes reduced the proportion of α-SMA-positive myofibroblasts and led to a thinner scar tissue with less collagen deposition *in vivo* (Fig. 8**i-l;** Additional file 1: S15g).

## Discussion

In clinical practice, it is essential to explore safe, cost-effective, and effective intervention medications to prevent HS pathogenesis and reduce the economic burden of its management. To achieve the above goals, we first conducted a prospective, multicenter, double-blind RCT study to assess the efficacy of 0.05% tretinoin cream in preventing HS formation. Based on a combination of subjective and objective evaluations, we found that 0.05% tretinoin cream was non-inferior to the first-line medication, silicone gel, in reducing HS formation, particularly in decreasing scar hyperplasia, myofibroblast activation, and collagen deposition. Several studies have investigated the effects of topical tretinoin cream on HS formation. Taheri *et al*. [41] conducted a retrospective study and discovered that the combination of topical clobetasol and tretinoin can prevent scar formation; however, this study primarily focused on superficial partial-thickness burn ulcers and could not ascertain the isolated effects of tretinoin on preventing HS development. Kwon *et al.* [13] demonstrated that both topical silicone gel and tretinoin cream could prevent HS formation; however, the research enrolled a relatively small sample size and included various inhomogeneous incisions, with only the modified VSS used to assess scar development. The insufficient number of participants and the narrow range of evaluation criteria might compromise the accuracy of the HS assessment. Most importantly, the two aforementioned studies were not RCTs, resulting in a lower level of evidence.

Our multicenter RCT addressed the shortcomings of previous studies and provided compelling clinical and mechanistic evidence to support the translational potential of tretinoin-based prevention of HSs, integrating insights from RCT and laboratory research. Notably, in comparison to the previously applied 0.025% tretinoin cream, the 0.05% tretinoin cream not only maintained a similar effect in reducing the overall incidence of HS, but also demonstrated a greater capacity for reducing scar thickness [13]. Furthermore, our findings underscore that the 0.05% tretinoin cream notably suppressed HSF proliferation and myofibroblast activation, potentially accounting for its superior efficacy in reducing scar thickness compared to silicone gel. Recognizing that extended follow-up periods can lead to elevated dropout rates, our RCT was designed with a follow-up period of 3 months, which may be considered relatively brief. Indeed, future studies with follow-up times of 6 or 12 months would provide a more comprehensive understanding of the intervention’s long-term preventive effects and potential side effects. We acknowledge that the comprehensiveness of baseline and clinical data collection for the enrolled population in the present study could be further optimized. To address this limitation, we plan to incorporate a broader spectrum of the aforementioned variables in our subsequent large-scale clinical trials. This refined study design will not only enable a more granular characterization of the baseline demographic and clinical distribution of the study cohort but also facilitate a rigorous investigation into the potential correlations between these variables and the therapeutic efficacy of HS treatments.

There is an increasing body of evidence suggesting that the “Warburg effect” promotes the FTM in multiple fibrotic diseases [42], [43]. In our study, we initially observed that HSFs undergo “Warburg effect”-like metabolic alterations, characterized by enhanced glycolysis and decreased OXPHOS, which are essential for TGF-β1-induced myofibroblast activation. In addition, in skin fibrotic diseases, TGF-β-stimulated glycolysis is characterized by the deposition of an abundant extracellular matrix (ECM), fibroblast proliferation, and the activation of myofibroblasts. Conversely, inhibiting glycolysis suppresses the FTM transition and the mRNA and protein levels of ECM-associated genes, including *Col-1*, fibronectin (*FN*), *α-SMA*, plasminogen activator inhibitor 1 (*PAI-1*), and connective tissue growth factor (*CTGF*) [44], [45]. The above research findings on aerobic glycolysis and skin fibrosis were consistent with our observations in HSFs, suggesting that targeting glucose metabolism in myofibroblasts may serve as a novel intervention strategy for attenuating HS formation and skin fibrosis.

Based on the abnormal glucose metabolic reprogramming in HSFs, our study elucidates novel mechanistic underpinnings of ATRA’s anti-fibrotic action in HS pathogenesis, providing a theoretical foundation for the clinical application of ATRA. Through integrated multi-omics profiling and functional metabolic assays, we demonstrate that ATRA inhibits myofibroblast activation *in vivo* and *in vitro* via metabolic reprogramming characterized by glycolysis inhibition and gluconeogenesis activation. Prior investigations have documented ATRA’s anti-fibrotic effects through inducing MerTK cleavage in liver fibrosis, decreasing Fos-related antigen 2 (Fra2)-mediated collagen accumulation in systemic sclerosis, and repressing the collagen synthesis and cross-linkages to restore ECM homeostasis in pancreatic fibrosis [8], [46], [47]. In contrast to previous findings, our research not only definitively identified HSFs as the effector cells for ATRA treatment during HS formation but also innovatively revealed the metabolic shift from aerobic glycolysis to OXPHOS as the primary response mechanism induced by ATRA in HSFs. Furthermore, the elevated glucose production and increased phosphoenolpyruvate carboxykinase (PEPCK, a gluconeogenic enzyme) expression following ATRA treatment suggested that ATRA can stimulate the gluconeogenic potential in HSFs. Existing studies have revealed that the liver and kidneys are the primary gluconeogenic organs in the body [48], [49]. However, it is now understood that gluconeogenesis also occurs in various organs, including the intestine and brain, as well as in tumors and fibrotic diseases, in response to exogenous stimuli such as salvianolic acid C and cortisol [48], [50], [51], [52], but the roles of gluconeogenesis in modulating HS pathogenesis have not yet been established. Our study addresses this knowledge gap and provides hitherto undocumented evidence of ATRA’s ability to enhance gluconeogenesis in HSs, while concurrently suppressing aerobic glycolysis, reducing intracellular lactate accumulation, diminishing the expression of critical glycolytic enzymes, and inhibiting HSF proliferation and myofibroblast activation. Building upon the above findings, it can be inferred that ATRA possesses significant therapeutic potential for skin fibrotic diseases with abnormal glucose metabolism. The combinatorial targeting of aerobic glycolysis suppression and gluconeogenic activation may represent a novel therapeutic paradigm for inhibiting the onset and progression of skin fibrotic diseases, particularly in the context of HS formation.

Furthermore, our study identified HIC1, PCK1, and PCK2 as key downstream functional molecules involved in the metabolic reprogramming of HSFs mediated by ATRA. *HIC1* has been reported as a tumor-suppressor gene; downregulation or loss of *HIC1* has been associated with the activation of CAFs, as well as increased tumor growth, invasion, and recurrence [53], [54]. Recent evidence highlights the modulating roles of HIC1 in lineage plasticity and skin reconstruction. Specifically, HIC1-lineage skin mesenchymal progenitors (MPs) and their progeny serve as the origin of regenerative fibroblasts during wound healing, and HIC1^+^ fibroblasts can migrate to the regenerative domain in wounds to facilitate neodermis formation [55]. Interestingly, *HIC1* deletion was found to increase fibroblast density within the regenerative domain, indicating that HIC1 can maintain wound microenvironment homeostasis by restricting the excessive proliferation of MPs and fibroblasts. Besides, HIC1^+^ fibroblasts exhibited higher chromatin accessibility of RARs and activation of RA signaling, while the mechanisms by which HIC1 inhibited fibroblast proliferation and its interactions with RA signaling remain unclear. In the present study, we reveal a novel mechanism by which ATRA induces *HIC1* overexpression in HSFs, thereby inhibiting fibroblast proliferation and myofibroblast activation through a reduction in aerobic glycolysis. We also identified the HIC1/TGF-β/glycolysis axis as a new pathway in regulating glycolysis. These findings substantiate that HIC1 acts as a downstream effector of ATRA-mediated RA signaling activation, as well as bridging a gap in understanding the mechanisms by which HIC1 regulates fibroblast proliferation and myofibroblast activation.

In addition to HIC1, ATRA treatment also leads to the upregulation of two distinct isoforms of PEPCK, namely PCK1 and PCK2, involved in glucose metabolism in HSFs. Growing evidence highlights the complex regulatory functions of PEPCK on gluconeogenesis [56]. Normally, PEPCK primarily regulates the initial rate-limiting step in hepatic gluconeogenesis, facilitating the conversion of non-carbohydrate substrates, such as lactate, into glucose, thereby maintaining glucose homeostasis [57]. Pathologically, PEPCK functions in various fibrotic diseases. Specifically, PCK1 enhances mitochondrial function and mitigates renal fibrosis by suppressing HK2, thereby blocking excessive glycolysis; conversely, PCK2 and lin-28 homolog A (LIN28A) synergistically promote pathological hypertrophy and fibrosis in cardiac tissue by increasing aerobic glycolysis levels [57], [58]. Accordingly, determining the regulatory effects of upregulated PCK1/PCK2 following ATRA treatment on aerobic glycolysis is crucial for understanding the role of PEPCK in skin fibrotic diseases. Through *in vitro* experiments, we first revealed a synergistic effect on PCK1 and PCK2 expression. Upregulation of either PCK1 or PCK2 in HSFs promotes gluconeogenesis while concurrently reducing aerobic glycolysis, lactate accumulation, and the expression of key glycolytic enzymes. These findings suggest that the gluconeogenesis induced by ATRA influences the fate of HSFs and contributes to the attenuation of scar formation.

Although we have established that HIC1, PCK1, and PCK2 are critical downstream molecules of ATRA-induced RA signaling, the primary regulatory mechanism remains elusive. Activation of RA signaling has been intricately linked to the remodeling of glucose metabolism and myofibroblast activation in both tumor and fibrotic diseases [59], [60]. Previous study established that the RARγ-dependent activation of the ATRA/RA signaling pathway significantly influences the differentiation trajectory of myofibroblasts, facilitating scarless wound healing [10]. In this study, we found that activating RARα in HSFs could substantially upregulate the expression of HIC1, PCK1, and PCK2, while activation of RARβ and RARγ only moderately elevated the expression of HIC1 or PCK2 with relatively lower fold-changes. As key functional molecules downstream of ATRA, HIC1, PCK1, and PCK2 are all crucial for the regulation of glucose metabolic homeostasis, as well as the modulation of proliferation and activation in HSFs. Based on the different responses of HIC1, PCK1, and PCK2 to the activation or inhibition of the aforementioned RARs, our research mainly revealed a role for RARα-dependent transcriptional activation of HIC1, PCK1, and PCK2 in HSFs in mediating ATRA’s effects on mitigating the pathogenesis of HSs. Nevertheless, further investigation is warranted to elucidate the effects of ATRA-activated RARβ and RARγ on reducing skin fibrosis, for the activation of RARβ and RARγ may moderately increase the expression of HIC1 or PCK2. Therefore, this work reveals a novel mechanism by which RARα-dependent upregulation of HIC1, PCK1, and PCK2 following ATRA treatment reduces fibroblast proliferation and myofibroblast activation by reprogramming glucose metabolism.

Through both *in vivo* and *in vitro* experiments, we found that tretinoin cream significantly reduced scar formation without impeding wound healing across various hypertrophic scarring models, underscoring the safety and efficacy of tretinoin cream as a medication. Furthermore, we also found that the upregulation of HIC1, PCK1, and PCK2 expression significantly reduces myofibroblast activation, collagen deposition, and scar hyperplasia, suggesting that HIC1, PCK1, and PCK2 are potential therapeutic targets for inhibiting skin fibrosis, particularly in the context of hypertrophic scarring. Given the intricate nature of HS development in humans, influenced by a variety of internal and external factors, our study was constrained by the relatively limited number of HS tissues and cells obtained from various patients for more mechanistic exploration. Therefore, future research necessitates more diverse sources and an adequate sample size of HSs to identify additional downstream molecules and functional pathways associated with ATRA treatment.

Tretinoin cream is a well-established and safe topical medication that is extensively used for the treatment of various skin disorders, with documented evidence of tolerable side effects [11], [61]. Similarly, a notable limitation in its application to healed wounds is the high prevalence of side effects such as skin burning, dryness, erythema, and desquamation, which affects over half of patients and may adversely impact patient compliance and the wider clinical adoption of tretinoin cream [62], [63]. To improve tolerability and mitigate side effects of topical tretinoin cream, several approaches have been explored, including short-contact therapy and new vehicle formulations, to enhance treatment adherence and overall satisfaction [64]. Specifically, recommendations include tretinoin cream for a brief duration of 30–60 min during the initial 2–4 weeks, along in conjunction with a moisturizing cream or lotion to enhance tolerability [65]. Alternatively, tretinoin cream may be applied every other day to minimize the frequency of exposure [66]. Optimizing the formulation and dosage of the vehicle is crucial for achieving a controlled release of ATRA at its specific site of action [67]. Polymeric emulsion technology has been utilized to facilitate the simultaneous release of ATRA with emollients and humectants, potentially enabling lower drug concentrations while maintaining efficacy [68]. Upon the onset of intolerable side effects, patients should promptly discontinue the medication, inform their physician, and apply a moisturizing cream or lotion topically. If the discomfort persists, they should seek timely consultation from a dermatologist. However, given its strong preventive effects, high biosafety, and significantly lower cost, 0.05% tretinoin cream holds promise as an innovative option for postoperative scar prevention.

## Conclusions

In conclusion, this study provides robust clinical evidence supporting the use of 0.05% tretinoin cream as a cost-effective alternative for preventing the formation of HSs; what’s more, we uncover a novel mechanism whereby metabolic reprogramming governs myofibroblast activation and fibroblast proliferation in HSs: RARα-dependent upregulation of HIC1, PCK1, and PCK2 in HSFs underpins ATRA’s anti-fibrotic effects, establishing these molecules and glucose metabolism as promising candidate therapeutic targets for skin fibrotic diseases (Additional file 1: Fig. S16). Notably, ATRA can serve as a novel dual modulator of glucose metabolism, offering a potential therapeutic option for glycometabolism-related diseases characterized by abnormal increases in aerobic glycolysis and impaired gluconeogenesis. Consequently, our work not only provides the practical insights and theoretical foundations for the application of tretinoin cream in attenuating HS formation, but also identifies new therapeutic options and targets for the treatment of glycometabolism-related skin fibrosis diseases.

## Abbreviations

AAV-HIC1: AAV-CMV-loxP-stop-loxP-*HIC1*

AAV-PCK1: AAV-CMV-loxP-stop-loxP-*PCK1*

AAV-PCK2: AAV-CMV-loxP-stop-loxP-*PCK2*

AAV-control: AAV-CMV-loxP-stop-loxP

ATRA: All-trans retinoic acid

ARD: Absolute risk difference

Col-1: Collagen type I

Col-3: Collagen type III

CRC: Clinical research coordinator

DEGs: Differentially expressed genes

DEMs: Differentially expressed metabolites

2-DG: 2-deoxy-D-glucose

ECM: Extracellular matrix

ECAR: Extracellular acidification rate

FTM: Fibroblast-to-myofibroblast

GO: Gene Ontology

HE: Hematoxylin and eosin

HIC1: Hypermethylated in cancer 1

HS: Hypertrophic scar

HSFs: Hypertrophic scar fibroblasts

IF: Immunofluorescence

KEGG: Kyoto Encyclopedia of Genes and Genomes

LSCI: Laser Speckle Contrast Imaging

NSFs: Fibroblasts from normal skin

OCR: Oxygen consumption rate

OXPHOS: Oxidative phosphorylation

PBS: Phosphate-buffered saline

PCA: Principal component analysis

PCK2: Phosphoenolpyruvate carboxykinase

POSAS: Patient and Observer Scar Assessment Scale

RCT: Randomized controlled trial

RARs: Retinoic acid receptors

RT-qPCR: Quantitative reverse transcription PCR

shRNA: Short hairpin RNA

α-SMA: α-smooth muscle actin

SEI: Scar elevation index

TGF-β: Transforming growth factor-β

VSS: Vancouver Scar Scale

## Ethics approval and consent to participate

This study was approved by the Ethics Committee of Xijing Hospital (KY20232367-F-1) and was conducted in accordance with the principles outlined in the Declaration of Helsinki. The trial was registered with the Chinese Clinical Trial Registry (https://www.chictr.org.cn, registration number: ChiCTR2500097242). All animal experiments were approved by the Medical Ethics Committee of the Fourth Military Medical University (IACUC20241264).

## Funding

This work was supported by the National Natural Science Foundation of China (82372530, 82072182), the Special Project of Xijing Hospital Clinical Research (XJZT24LZ14), and the Intramural Research Program Project founded by Fourth Military Medical University (2024QMJJ014).

## Data Availability

All data that support the findings of this study are available from the corresponding authors upon reasonable request. The RNA-seq data have been deposited in the NCBI SRA database (SRA accession: PRJNA1205786).

## Footnotes

Appendix A Supplementary data associated with this article can be found in the online version at doi:10.1016/j.mmr.2026.100021.
