# Supplementary material for: From RCT to mechanistic study: ATRA reverses myofibroblast activation by reprogramming glucose metabolism via HIC1 and PCK1/2 to attenuate hypertrophic scar formation
Source: Mil Med Res. 2026 Apr 27;13(1):100021. doi: 10.1016/j.mmr.2026.100021 (PMC13138214; doi:10.1016/j.mmr.2026.100021)
Supplement: Supplementary file 1 — Supplementary material [file mmc1.pdf]

Materials and methods

Source and identification of animals

Balb/C mice (8–10 weeks old, female) and New Zealand White rabbits (2.5–3.0 kg, female) were obtained from the Experimental Animal Center of the Fourth Military Medical University. C57BL/6J Col1-CreER mice (8 weeks old, male) were purchased from Cyagen Biosciences Inc. (China). To generate experimental cohorts, male C57BL/6J Col1-CreER mice were bred with female wild-type C57BL/6J mice. Genotypes of Col1a2-CreER mice were confirmed by PCR amplification of genomic DNA extracted from mouse tail biopsies, using a one-step mouse genotyping kit (PD101-01, Vazyme, China). All animal experiments were approved by the Medical Ethics Committee of the Fourth Military Medical University (IACUC20241264).

Histological analysis of scar tissue

The wound healing and scar formation abilities were evaluated through histological analysis of excised scars obtained from human subjects, mouse dorsal skin, and rabbit ears. Hematoxylin and eosin (HE) staining was used to assess the general morphology of the scar tissue, while Masson's trichrome staining was employed to evaluate collagen deposition. For tissue sections, immunofluorescence (IF) staining was performed overnight at 4 °C using primary antibodies targeting hypermethylated in cancer 1 (HIC1), phosphoenolpyruvate carboxykinase 1/2 (PCK1/ PCK2), and α-smooth muscle actin (α-SMA) (**Additional file 1: Table S3**). This was followed by incubation with fluorescently labeled secondary antibodies and DAPI for nuclear staining.

HE staining

HE staining was performed according to standard histological protocols. Briefly, formalin-fixed, paraffin-embedded tissue sections (4–5 μm) were deparaffinized in xylene and rehydrated through a graded ethanol series to distilled water. Nuclei were stained with Harris's hematoxylin for 3–8 min, followed by rinsing in running tap water to remove excess dye. The sections were then treated with 1% acid ethanol for a few seconds to remove non-specific nuclear staining and subsequently blued in 0.2% ammonia water to optimize nuclear contrast. After a further rinsing in distilled water, the cytoplasm and extracellular matrix were counterstained with eosin Y solution for 30 s to 2 min. The stained sections were rapidly dehydrated through a graded series of alcohols, cleared in xylene, and finally

mounted with a synthetic resinous medium for microscopic examination.

Masson's trichrome staining

Masson's trichrome staining was conducted to differentially visualize collagen fibers, muscle, and erythrocytes. Following deparaffinization and rehydration as described for HE, tissue sections were immersed in Bouin's fixative and incubated at 56 °C for 1 h, then cooled and rinsed under running tap water until the yellow colour disappeared. Nuclei were firstly stained with Weigert's iron hematoxylin for 5–10 min, followed by thorough washing. The sections were then treated with Biebrich scarlet-acid fuchsin solution for 5–10 min to stain cytoplasm, muscle, and erythrocytes. Without rinsing, the slides were directly placed into a phosphomolybdic-phosphotungstic acid solution for 5–10 min, which differentially removes the red dye from collagens. Subsequently, the sections were transferred directly to aniline blue solution for 2–5 min, staining the decolorized collagen fibers blue. After a brief rinse in distilled water, slides were treated with 1% acetic acid for 1–2 min, dehydrated rapidly in absolute alcohol, cleared in xylene, and mounted with a permanent mounting medium.

RNA isolation and quantitative real-time PCR

Total RNA from hypertrophic scar fibroblasts (HSFs) was extracted using TRIzol reagent (Invitrogen, USA), and complementary DNA (cDNA) was synthesized using the PrimeScript RT Master Mix (TaKaRa Biotechnology, China) according to the manufacturer's instructions. Quantitative reverse transcription PCR (RT-qPCR) was performed using SYBR-Green qPCR Master Mix (TaKaRa Biotechnology, China) to determine the expression levels of target genes. Gene-specific primers used in this study are listed in **Additional file 1: Table S11**. Relative gene expression was calculated using the $2^{-\Delta\Delta Ct}$ method, with *β-actin* as the internal reference for normalization.

Scratch wound healing assay

Cell migration was assessed using a scratch wound healing assay, as previously described [1]. When HSFs cultured in 6-well plates reached 90% confluence, a sterile 200 μl pipette tip was used to create a linear scratch in the monolayer. The culture medium was then replaced with 3% Fetal bovine serum-Dulbecco's modified Eagle medium (FBS-DMEM) to minimize the influence of cell proliferation. At 24 and 36 h post-scratch, cells were washed with phosphate-buffered saline (PBS), and images were captured under a microscope. The wound closure area was quantified using ImageJ software to evaluate the migration capacity of HSFs in different treatment groups.

Transwell assay

The Transwell assay was conducted as previously described [2]. HSFs were seeded into Transwell-24 well plates (Corning, USA) with 8 μm pore filters. DMEM with 10% FBS was added to the lower chamber, while the upper chamber contained 1×10^4 HSFs suspended in DMEM with 2% FBS. After a 36 h-treatment at 37 °C, HSFs that migrated to the underside of the membrane were fixed with 4% paraformaldehyde (Sigma, USA), stained with 1% crystal violet (MedChemExpress, USA), and then counted using ImageJ software.

Collagen gel contraction assay

The fibroblast-mediated collagen contraction assay was performed using the Collagen Contraction Assay kit (Cell Biolabs, USA) according to the manufacturer's protocol. Briefly, 5×10^5 HSFs and 500 μl of collagen preparation were mixed, and the mixture was added to each well of a 24-well plate. Images were captured at 0, 24, and 36 h after the detachment of collagen gels, and the relative contraction rates were analyzed using ImageJ software.

Cell cycle analysis

HSFs were collected 48 h after seeding, washed with ice-cold PBS, and fixed with ice-cold 70% ethanol overnight. After the ethanol was removed, HSFs were stained with propidium iodide (PI) (BD Biosciences, USA) and RNase (BD Biosciences, USA) for 30 min at room temperature in the dark.

Apoptosis assay

HSFs were collected and subjected to staining using the Annexin V/FITC Kit (BD Biosciences, USA), following the manufacturer's instructions. Flow cytometry analysis (Beckman Coulter, USA) was performed to detect the cell cycle and apoptosis.

Western blotting analysis

Western blotting assay was performed as previously described [3]. Scar tissues and isolated HSFs were lysed in ice-cold RIPA lysis buffer (CWbio, China) supplemented with 1% protease and phosphatase inhibitors (Sigma, USA) and 1 mmol/L phenylmethylsulfonyl fluoride (PMSF) (Yeasen, China). After centrifugation at 12,000×*g* for 15 min at 4 °C, the supernatant was collected, and protein concentrations were determined using the bicinchoninic acid (BCA) assay (Beyotime, China). Proteins were separated

by SDS-PAGE, transferred onto PVDF membranes, and incubated with primary antibodies at 4 °C overnight. The primary antibodies used include anti-β-actin, anti-HIC1, anti-PCK1, anti-PCK2, anti-transforming growth factor-β1 (TGF-β1), anti-α-SMA, anti-collagen type I, and anti-collagen type III (**Additional file 1: Table S3**). After washing, membranes were incubated with HRP-conjugated goat anti-rabbit or goat anti-mouse secondary antibodies for 2 h at room temperature. Protein bands were visualized using a chemiluminescent detection reagent (Cell Biosciences, USA), and band intensities were quantified using ImageJ software.

Transcriptome and metabolome sequencing

HSFs obtained from 4 patients were treated with 50 μg/ml all-trans retinoic acid (ATRA; HY-14649, MedChemExpress, USA) or PBS for 48 h at 60% confluence (**Additional file 1: Table S4**). Following treatment, the digested HSFs were subjected to transcriptome and metabolome sequencing.

RNA extraction and library construction for transcriptome sequencing

Total RNA was extracted from HSFs using TRIzol reagent (Invitrogen, USA) following the manufacturer's instructions. RNA quality was assessed using the 5300 Bioanalyzer (Agilent, USA) and quantified with a Nanodrop ND-2000 (Thermo Fisher Scientific, USA). RNA purification, reverse transcription, library construction, and sequencing were performed at Shanghai Majorbio Bio-pharm Biotechnology Co., Ltd. (Shanghai, China) following the manufacturer's guidelines (Illumina, USA). The RNA-sequencing (RNA-seq) transcriptome library was prepared using the Illumina Stranded mRNA Prep Ligation kit (San Diego, USA). Double-stranded cDNA was synthesized using a Superscript double-stranded cDNA synthesis kit (Invitrogen, USA), followed by end-repair, phosphorylation, and "A" base addition according to the standard library construction protocol. The final cDNA library was quantified using a Qubit 4.0 fluorometer and sequenced on the Illumina Novaseq 6000 platform using a paired-end strategy.

Differential expression and functional enrichment in the transcriptome

Following RNA-seq experiments, principal component analysis (PCA) was performed to assess variability both between the PBS- (Control) and ATRA-treated (Treat) groups, and within the aforementioned groups. Differentially expressed genes (DEGs) between ATRA-treated and PBS-treated HSFs were identified by calculating transcript expression levels using the transcripts per million reads (TPM) method. Gene abundances were quantified using RSEM (http://deweylab.biostat.wisc.edu/rsem/) [4]. To determine the functional significance of DEGs, Gene

Ontology (GO) and Kyoto Encyclopedia of Genes and Genomes (KEGG) enrichment analyses were conducted. GO terms and KEGG pathways with a *P*-value<0.05 were considered significantly enriched.

Metabolite extraction and liquid chromatography-tandem mass spectrometry (LC-MS/MS) analysis

Metabolite extraction was performed as previously described [5]. Briefly, 200 μl cell suspension was mixed with 0.02 mg/ml of an internal standard (L-2-chlorophenylalanine) and 300 μl of an acetonitrile/methanol solution (1:1, v/v). The mixtures were vortexed for 1 min and incubated at -20 °C for 30 min to facilitate protein precipitation. After centrifugation at 13,000×*g* for 15 min at 4 °C, the supernatant was transferred to MS vials for subsequent LC-MS/MS analysis. The LC-MS/MS analysis was performed on a UHPLC-Q Exactive HF-X system (Thermo, USA) equipped with an ACQUITY HSS T3 column (100 mm×2.1 mm, 1.8 μm; Waters, USA).

The mobile phase consisted of solvent A (0.1% formic acid in water:acetonitrile=95:5, v/v) and solvent B (0.1% formic acid in acetonitrile:isopropanol:water=47.5:47.5:5, v/v). The column temperature was maintained at 4 °C, and the injection volume was 3 μl. Mass spectrometry was performed using an electrospray ionization (ESI) source operating in both positive and negative modes. The mass resolution was set to 60,000 for full MS and 7500 for MS/MS resolution. Data acquisition was conducted in the Data Dependent Acquisition (DDA) mode. Raw LC-MS data were processed using Progenesis QI software (Waters Corporation, USA), and metabolites were manually verified using databases including the Human Metabolome Database (HMDB) (http://www.hmdb.ca/), Metlin (https://metlin.scripps.edu/), and the Majorbio Database (http://www.i-sanger.com/). PCA was performed using the R package "ropls" (version 1.6.2). Differentially expressed metabolites (DEMs) between groups were mapped to biochemical pathways using KEGG (http://www.genome.jp/kegg/) for metabolic enrichment and pathway analysis.

Glucose uptake assay

HSFs were seeded into 24-well plates and cultured to 80% confluence at 37 °C. After overnight incubation, the media was removed, and the cells were thoroughly washed with PBS. HSFs were then incubated in glucose-free DMEM (Hyclone, USA) containing 100 μmol/L 2-deoxy-2-[7-nitro-2,1,3-benzoxadiazol-4-yamino]-D-glucose (2-NBDG) (Thermo, USA) at 37 °C with 5% CO_2 for 1 h. After incubation, cells were digested, washed twice with PBS, and glucose uptake was measured by flow cytometry (BD Accuri C6, BD Biosciences, USA).

ATP production assay

Cellular ATP levels were measured using the ATP Assay Kit (ab83355, Abcam, USA) (**Additional file 1: Table S12**). Briefly, HSFs cultured in 6-well plates were harvested, washed, and resuspended in 100 μl of ATP assay buffer. The cells were then homogenized, and cellular debris was removed by centrifugation at 12,000× *g* for 10 min at 4 °C. The supernatants were collected, and the deproteinized samples were incubated with the ATP probe following the manufacturer's instructions. Absorbance was measured at 560 nm using a microplate reader (BioTek, USA).

Lactate production assay

Cellular lactate levels were measured using a lactate assay kit (JL-T1068, Jianglai Bio, China) (**Additional file 1: Table S12**). Briefly, HSFs cultured in a 6-well plate were harvested, homogenized, and centrifuged at 12,000×*g* for 10 min at 4 °C. The supernatants were collected, and the cellular lactate production was quantified according to the manufacturer's instructions. Absorbance was measured at 450 nm using a microplate reader (BioTek, USA).

Glucose production assay

The glucose production assay was performed as previously described [6]. Briefly, HSFs in different groups were washed twice with PBS, followed by stimulation with 2 mmol/L sodium pyruvate (Sigma-Aldrich, USA) and 20 mmol/L sodium lactate (Sigma-Aldrich, USA) in glucose- and serum-free DMEM medium (Hyclone, USA) for 6 h. After incubation, the cell culture medium was centrifuged, and the supernatants were collected. The glucose concentration was quantified using the Glucose Assay kit (GAGO20-1KT, Sigma-Aldrich, USA) according to the manufacturer's instructions.

Seahorse assay

The oxygen consumption rate (OCR) and extracellular acidification rate (ECAR) in HSFs were measured using the Seahorse XFe24 Flux Analyzer (Agilent Technologies, USA) according to the manufacturer's protocols [7]. Briefly, HSFs were seeded onto pre-collagen-coated Seahorse XFe24 well plates at a density of 2×10^4 cells per well and incubated overnight. For OCR measurements, the basal OCR was recorded, followed by sequential treatment with 1 μmol/L oligomycin A, 1 μmol/L carbonyl cyanide 4-(trifluoromethoxy) phenylhydrazone (FCCP), and 1 μmol/L rotenone/antimycin A. Similarly, ECAR was measured by first recording the basal ECAR, followed by sequential treatment with 10 mmol/L glucose, 1 μmol/L oligomycin, and 50 mmol/L 2-deoxy-D-glucose (2-DG). The values obtained were normalized to the number of cells in each well at the end of the assay and analyzed using

the Seahorse Wave software.

Chromatin immunoprecipitation (ChIP)-qPCR assay

The ChIP-qPCR assay was conducted as previously described [8]. Briefly, 1×10^8 HIC1- or retinoic acid receptor alpha (RARα)-overexpressing HSFs were cross-linked with 1% formaldehyde. The cell lysate was then sonicated to fragment the DNA into fragments of 200–500 bp for ChIP-qPCR analysis. Immunoprecipitation was performed using antibodies against normal immunoglobulin G (IgG), HIC1, and anti-RARα (**Additional file 1: Table S3**). RNase A and proteinase K were added to digest protein-DNA complexes, and the precipitated DNA fragments were subsequently analyzed by qPCR. The specific primers used for ChIP-qPCR are provided in **Additional file 1: Table S11**.

Luciferase reporter assay

Luciferase reporter assays were performed as previously described [9]. The target sequences of *TGF-β1*, *HIC1*, *PCK1*, and *PCK2* promoters were amplified, digested, and cloned into the PGL3 basic vector (GeneCreat, China). The schematic diagram of the firefly luciferase reporters is shown in **Additional file 1: Fig. S10**. The human embryonic kidney cells (293T, TCH-C101) were bought from HyCyte™, Suzhou, China. These reporter vectors were transfected into HSFs or 293T cells using Lipofectamine 3000 (L3000015, Invitrogen, USA) following the manufacturer's instructions. Firefly luciferase activity was detected using a luciferase assay kit (RG027, Beyotime, China) with the microplate reader (BioTek, USA). The relative luciferase activity was normalized to Renilla luciferase activity.

Refencences

1. Li X, Zhang Z, Wang L, Zhao H, Jia Y, Ma X, *et al*. Three-phase extraction of polysaccharide from stropharia rugosoannulata: process optimization, structural characterization and bioactivities. Front Immunol. 2022;13:994706.
2. Di Francesco D, Bertani F, Fusaro L, Clemente N, Carton F, Talmon M, *et al*. Regenerative potential of a bovine ECM-derived hydrogel for biomedical applications. Biomolecules. 2022;12(9);1222.
3. Gao Y, Zhou J, Xie Z, Wang J, Ho CK, Zhang Y, *et al*. Mechanical strain promotes skin fibrosis through LRG-1 induction mediated by ELK1 and ERK signalling. Commun Biol. 2019;2:359.
4. Thakur V. RNA-seq data analysis for differential gene expression using HISAT2-StringTie-ballgown pipeline. Methods Mol Biol. 2024;2812:101-13.
5. Zhu Y, Ye J, Qin P, Yan X, Gong X, Li X, *et al*. Analysis of serum reproductive hormones and ovarian genes in pubertal female goats. J Ovarian Res. 2023;16(1):69.
6. Ekberg K, Landau BR, Wajngot A, Chandramouli V, Efendic S, Brunengraber H, *et al*. Contributions by kidney and liver to glucose production in the postabsorptive state and after 60 h of fasting. Diabetes. 1999;48(2):292-8.
7. Qian W, Van Houten B. Alterations in bioenergetics due to changes in mitochondrial DNA copy number. Methods. 2010;51(4):452-7.
8. Dai W, Wu J, Peng X, Hou W, Huang H, Cheng Q, *et al*. CDK12 orchestrates super-enhancer-associated ccdc137 transcription to direct hepatic metastasis in colorectal cancer. Clin Transl Med. 2022;12(10):e1087.
9. Zhang J, Liu B, Xu C, Ji C, Yin A, Liu Y, *et al*. Cholesterol homeostasis confers glioma malignancy triggered by hnRNPA2B1-dependent regulation of SREBP2 and LDLR. Neuro Oncol. 2024;26(4):684-70.

Table S1 Modified Vancouver Scar Scale (VSS) components and parameters used in the study

**Skin characteristics**	**Parameters**
Scar height	
0	Flat
1	<2 mm
2	2–5 mm
3	>5 mm
Pliability	
0	Normal
1	Supple-flexible with minimal resistance
2	Yielding, giving way to pressure
3	Firm, inflexible, not easily moved, resistant to manual pressure
4	Banding-rope-like tissue that blanches with the extension of the scar
5	Contracture, permanent shortening of scar, producing deformity or distortion
Vascularity	
0	Normal
1	Pink
2	Red
3	Red to purple
4	Purple
Pigmentation	
0	Normal
1	Hypo-pigmentation
2	Hyper-pigmentation
3	Mixed pigmentation

Table S2 Patient and Observer Scar Assessment Scale (POSAS) components, categories, and parameters used in the study

Skin characteristics	Parameters
POSAS observer scale	
Vascularity (Pale; pink; red; purple; mix)	1=normal skin<br>10=worst scar imaginable
Pigmentation (Hypo; hyper; mix)	
Thickness (Thicker; thinner)	
Relief (More; less; mix)	
Pliability (Supple; stiff; mix)	
Surface area (Expansion; contraction; mix)	
Overall opinion	
POSAS patient scale	
Has the scar been painful for the past few weeks?	1=no, not at all<br>10=yes, very much
Has the scar been itching for the past few weeks?	
Is the scar colour different from the colour of your normal skin?	1=no, as normal skin<br>10=yes, very different
Is the stiffness of the scar different from your normal skin?	
Is the thickness of the scar different from your normal skin?	
Is the scar more irregular than your normal skin?	
Overall opinion	1=as normal skin 10=very different

Table S3 Antibodies used for Western blotting, IF, ICC, and ChIP

Experiment	Antibody	Dilution	Catalog No.	Manufacturer	Origin
Western blotting	Anti-HIC1	1:200	sc-271499	Santa Cruz	CA, USA
	Anti-PEPCK-C/PCK1	1:250	sc-373972	Santa Cruz	CA, USA
	Anti-PEPCK-M/PCK2	1:250	PA5-28078	Invitrogen	CA, USA
	Anti-TGF-β1	1:500	ab215715	Abcam	Cambridge, UK
	Anti-α-SMA	1:2000	14395-1-AP	Proteintech	Chicago, USA
	Anti-collagen type I	1:2000	14695-1-AP	Proteintech	Chicago, USA
	Anti-collagen type III	1:1000	22734-1-AP	Proteintech	Chicago, USA
	Anti-β-actin	1:1000	sc-517582	Santa Cruz	CA, USA
	Goat anti-rabbit secondary antibodies	1:5000	ab205718	Abcam	Cambridge, UK
	Goat anti-mouse secondary antibodies	1:5000	ab205719	Abcam	Cambridge, UK
IF	Anti-HIC1	1:50	sc-271499	Santa Cruz	CA, USA
	Anti-PEPCK-C/PCK1	1:100	sc-373972	Santa Cruz	CA, USA
	Anti-PEPCK-M/PCK2	1:100	PA5-28078	Invitrogen	CA, USA
	Anti-Ki67	1:500	ab15580	Abcam	Cambridge, UK
	Anti-α-SMA	1:1000	14395-1-AP	Proteintech	Chicago, USA
ICC	Anti-HIC1	1:100	sc-271499	Santa Cruz	CA, USA
	Anti-PEPCK-C/PCK1	1:80	sc-373972	Santa Cruz	CA, USA
	Anti-PEPCK-M/PCK2	1:100	PA5-28078	Invitrogen	CA, USA
	Anti-α-SMA	1:500	14395-1-AP	Proteintech	Chicago, USA
ChIP	Anti-HIC1	5 µg/100 µg protein	sc-271499	Santa Cruz	CA, USA
	Anti-RARA	2 µg/100 µg protein	10331-1-AP	Proteintech	Chicago, USA
	IgG	1 µg/µl	2729S	CST	MA, USA

IF. Immunofluorescence; ICC. Immunocytochemistry; ChIP. Chromatin immunoprecipitation; HIC1. Hypermethylated in cancer 1; PCK1. Phosphoenolpyruvate carboxykinase 1; PCK2. Phosphoenolpyruvate carboxykinase 2; TGF-β1. Transforming growth factor-β1; α-SMA. α-smooth muscle actin; RARα. Retinoic acid receptor alpha

Table S4 Demographic and clinical characteristics of volunteers for hypertrophic scar fibroblasts (HSFs) obtaining

Sample	Gender	Age (year)	BMI (kg/m^2)	Location	Diagnosis
1	Male	37	20.7	Trunk	Hypertrophic scar
2	Male	42	21.4	Extremity	Hypertrophic scar
3	Male	33	21.9	Trunk	Hypertrophic scar
4	Female	28	19.5	Trunk	Hypertrophic scar

BMI. Body mass index

Table S5 Incidence rates of hypertrophic scarring [% (90% CI)]

Type of analysis	Tretinoin	Silicone	ARD
ITT analysis	24.63 (14.75–34.51)	33.28 (22.81–43.74)	–8.65 (–23.03 to 5.74)
Sensitivity analysis	27.78 (15.83–39.72)	37.93 (25.44–50.42)	–10.15 (–27.43 to 7.13)

ARD. Absolute risk difference; ITT. Intent-to-treat

Table S6 Side effects according to the Medical Dictionary for safety analysis set [*n* (%)]

System organ classification	Silicone (*n*=58)	Tretinoin (*n*=54)	*P*-value
General disorders and the administration site			
Application site burning	9 (15.52)	20 (37.04)	0.009
Application site inflammation	0	2 (3.70)	0.230
Application site pruritus	10 (17.24)	11 (20.37)	0.672
Pain	5 (8.62)	10 (18.52)	0.124
Infections and infestations			
Eczema infected	0	0	-
Folliculitis	0	0	-
Injury, poisoning, and procedural complications			
Blister	0	2 (3.70)	0.230
Musculoskeletal and connective tissue disorders			
Arthralgia	0	0	-
Nervous system disorders			
Burning sensation	2 (3.45)	6 (11.11)	0.152
Skin and subcutaneous tissue disorders			
Alopecia	0	0	-
Dry skin	13 (22.41)	30 (55.56)	< 0.001
Eczema	0	0	-
Erythema	5 (8.62)	11 (20.37)	0.076
Pain in the skin	5 (8.62)	10 (18.52)	0.115
Pruritus	10 (17.24)	11 (20.37)	0.672
Psoriasis	0	0	-
Skin burning sensation	9 (15.52)	20 (37.04)	0.009
Skin fissure	0	0	-

"-" represents blank

Table S7 Severity grading of side effects for the safety analysis set [*n* (%)]

System organ classification	Silicone (*n*=58)			Tretinoin (*n*=54)		
	Mild	Moderate	Severe	Mild	Moderate	Severe
General disorders and the administration site						
Application site burning	8 (88.89)	1 (11.11)	0	17 (85.00)	3 (15.00)	0
Application site inflammation	-	-	-	2 (100)	0	0
Application site pruritus	9 (90.00)	1 (10.00)	0	11 (100)	0	0
Pain	4 (80.00)	1 (20.00)	0	10 (100)	0	0
Injury, poisoning, and procedural complications						
Blister	-	-	-	2 (100)	0	0
Nervous system disorders						
Burning sensation	2 (100)	0	0	4 (66.67)	2 (33.33)	0
Skin and subcutaneous tissue disorders						
Dry skin	10 (76.92)	3 (23.08)	0	24 (80.00)	6 (20.00)	0
Erythema	4 (80.00)	1 (20.00)	0	8 (72.73）	3 (27.27)	0
Pain of skin	4 (80.00)	1 (20.00)	0	10 (100)	0	0
Pruritus	9 (90.00)	1 (10.00)	0	11 (100)	0	0
Skin burning sensation	8 (88.89)	1 (11.11)	0	17 (85.00)	3 (15.00)	0

“-” represents blank

Table S8 Duration of the side effects for the safety analysis set (d, mean±SD)

	**Silicone (*n*=58)**	**Tretinoin (*n*=54)**
General disorders and the administration site		
Application site burning	4.67±1.58	5.00±1.86
Application site inflammation	-	6.00±1.41
Application site pruritus	4.90±1.79	6.45±1.70
Pain	4.80±1.48	4.80±1.62
Injury, poisoning, and procedural complications		
Blister	-	3.50±0.71
Nervous system disorders		
Burning sensation	4.50±2.12	4.50±1.52
Skin and subcutaneous tissue disorders		
Dry skin	5.00±2.04	9.20±3.37
Erythema	5.00±2.12	6.36±2.01
Pain in the skin	4.80±1.48	4.80±1.62
Pruritus	4.90±1.79	6..45±1.70
Skin burning sensation	4.67±1.58	5.00±1.86

"-" represents blank

Table S9 Remission and tolerance of the side effects for the safety analysis set [*n* (%)]

System organ classification	Remission & Tolerance	Group	Silicone (*n*=58)	Tretinoin (*n*=54)
General disorders and the administration site				
Application site burning	Remission modalities	Spontaneous remission	9 (100)	20 (100)
		Required intervention	0	0
	Tolerance	Yes	9 (100)	20 (100)
		No	0	0
Application site inflammation	Remission modalities	Spontaneous remission	-	2 (100)
		Required intervention	-	0
	Tolerance	Yes	-	2 (100)
		No	-	0
Application site pruritus	Remission modalities	Spontaneous remission	10 (100)	11 (100)
		Required intervention	0	0
	Tolerance	Yes	10 (100)	11 (100)
		No	0	0
Pain	Remission modalities	Spontaneous remission	5 (100)	10 (100)
		Required intervention	0	0
	Tolerance	Yes	5 (100)	10 (100)
		No	0	0
Injury, poisoning, and procedural complications				
Blister	Remission modalities	Spontaneous remission	-	2 (100)
		Required intervention	-	0
	Tolerance	Yes	-	2 (100)
		No	-	0
Nervous system disorders				
Burning sensation	Remission modalities	Spontaneous remission	2 (100)	6 (100)
		Required intervention	0	0
	Tolerance	Yes	2 (100)	6 (100)
		No	0	0

**System organ classification**	**Remission & Tolerance**	**Group**	**Silicone (*n*=58)**	**Tretinoin (*n*=54)**
Skin and subcutaneous tissue disorders				
Dry skin	Remission modalities	Spontaneous remission	13 (100)<br>30 (100)	
		Required intervention	0<br>0	
	Tolerance	Yes	13 (100)	30 (100)
		No	0	0
Erythema	Remission modalities	Spontaneous remission	5 (100)	11 (100)
		Required intervention	0	0
	Tolerance	Yes	5 (100)	11 (100)
		No	0	0
Pain of skin	Remission modalities	Spontaneous remission	5 (100)	10 (100)
		Required intervention	0	0
	Tolerance	Yes	5 (100)	10 (100)
		No	0	0
Pruritus	Remission modalities	Spontaneous remission	10 (100)	11 (100)
		Required intervention	0	0
	Tolerance	Yes	10 (100)	11 (100)
		No	0	0
Skin burning sensation	Remission modalities	Spontaneous remission	9 (100)	20 (100)
		Required intervention	0	0
	Tolerance	Yes	9 (100)	20 (100)
		No	0	0

Table S10 shRNA sequences of target genes

Gene	shRNA#1 (5'-3')	shRNA#2 (5'-3')
RARα	GAUGCUAAUGAAGAUUACU	GCAUCUACAAGCCUUGCUU
RARβ	GGCCUUACCCUAAAUCGAA	GACCUUGAGGAACCGACAA
RARγ	CUGUAUCGCAUAUUGCUGA	GCGUAUCUGCACAAGGUAC
HIC1	CCCUGGUGGUGCAUGACAA	CGUCGUGCGACAAGAGCUA
PCK1	GGAUGAAGUUUGACGCACA	CCAUUGAAGGCAUUAUCUU
PCK2	GAUGAGGUUUGACAGUGAA	CACGAGUAGAGAGCAAGAC

RARα. Retinoic acid receptor alpha; RARβ. Retinoic acid receptor beta; RARγ. Retinoic acid receptor gamma; HIC1. Hypermethylated in cancer 1; PCK1. Phosphoenolpyruvate carboxykinase 1; PCK2. Phosphoenolpyruvate carboxykinase 2

Table S11 Gene-specific primers used for qRT-PCR (human)

Gene	Forward primer sequence (5'-3')	Reverse primer sequence (5'-3')
RARα	AAGCCCGAGTGCTCTGAGA	TTCGTAGTGTATTTGCCCAGC
RARβ	TCCGAAAAGCTCACCAGGAAA	GGCCAGTTCACTGAATTTGTCC
RARγ	TGTCACCGCGACAAAAACTGT	CGAGGGGAAAGTCTCCTGA
HIC1	GTGATCATCGTGGTGCAGAA	CAGGTTGAGCAGGTTGTCAT
PCK1	TCCGAAAAGCTCACCAGGAAA	GGCCAGTTCACTGAATTTGTCC
PCK2	TGTCACCGCGACAAAAACTGT	CGAGGGGAAAGTCTCCTGA
GLUT	GGCCAAGAGTGTGCTAAAGAA	ACAGCGTTGATGCCAGACAG
GAPDH	AGAAGGCTGGGGCTCATTTG	AGGGGCCATCCACAGTCTTC
HK2	GAGCCACCACTCACCCTACT	CCAGGCATTCGGCAATGTG
LDHA	ATGGCAACTCTAAAGGATCAGC	CCAACCCCAACAACTGTAATCT
PFK	GCTGGGCGGCACTATCATT	TCAGGTGCGAGTAGGTCCG
PKM	ATGTCGAAGCCCCATAGTGAA	TGGGTGGTGAATCAATGTCCA
FBP1	TCTACCCGGTTCAAGCATGG	CAGCAATGCCATAGAGGTGC
G6PD	ACGACGAAGCGCAGACAG	TCCGACTGATGGAAGGCATC
TGF-β1	GGCCAGATCCTGTCCAAGC	GTGGGTTTCCACCATTAGCAC
α-SMA	CTATGAGGGCTATGCCTTGCC	GCTCAGCAGTAGTAACGAAGGA
Col-1	GTGCGATGACGTGATCTGTGA	CGGTGGTTTCTTGGTCGGT
β-actin	AGCGAGCATCCCCCAAAGTT	GGGCACGAAGGCTCATCATT

RARα. Retinoic acid receptor alpha; RARβ. Retinoic acid receptor beta; RARγ. Retinoic acid receptor gamma; HIC1. Hypermethylated in cancer 1; PCK1. Phosphoenolpyruvate carboxykinase 1; PCK2. Phosphoenolpyruvate carboxykinase 2; GLUT. Glucose transporter; GAPDH. Glyceraldehyde-3-phosphate dehydrogenase; HK2. Hexokinase 2; LDHA. Lactate dehydrogenase; PFK. 6-phosphofructokinase; PKM. Pyruvate kinase M; FBP1. Fructose-1,6-bisphosphatase 1; G6PD. Glucose-6-phosphate dehydrogenase; TGF-β1. Transforming growth factor-β1; α-SMA. α-Smooth muscle actin; Col-1. Collagen type I

Table S12 Kits in this study

Kit	Catalog No.	Manufacturer	Origin
BCA Protein Quantification Kit	P0398S	Beyotime	Shanghai, China
CCK-8 Cell Proliferation Assay Kit	C0037	Beyotime	Shanghai, China
EdU Kit	C0071S	Beyotime	Shanghai, China
Annexin V/FITC Kit	556547	BD	New Jersey, USA
Cell Cycle/DNA Kits	340242	BD	New Jersey, USA
Collagen Contraction Assay Kit	CBA-5020	Cell Biolabs	San Diego, USA
ATP Assay Kit	ab83355	Abcam	Cambridge, UK
Seahorse XF Cell Mito Stress Test Kit	103015-100	Agilent	California, USA
Glucose (GO) Assay Kit	GAGO20	Sigma-Aldrich	Taufkirchen, Germany
Lactic Acid Content Test Kit	JL-T1068	Shanghai JIANGLAI	Shanghai, China
Reverse Transcription Kit	RR047A	TaKaRa	Dalian, China
RT-qPCR Kit	CN830A	TaKaRa	Dalian, China
Chromatin Immunoprecipitation (ChIP) Kit	9003	CST	MA, USA
Dual-Luciferase Reporter Assay Kit	RG027	Beyotime	Shanghai, China

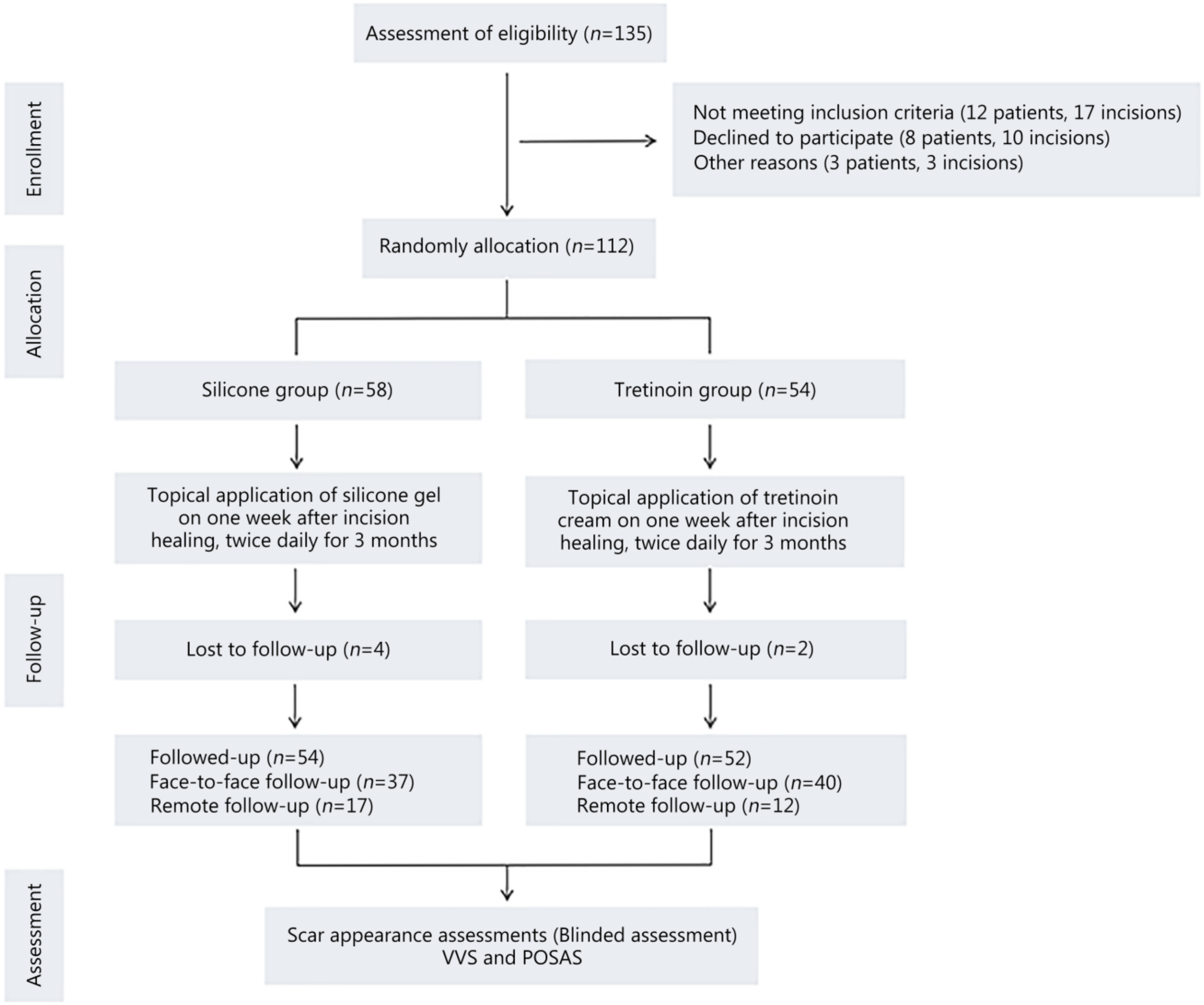

Fig. S1 Flowchart of the progression of participants and pipeline of the RCT study. RCT. Randomized controlled trial; VVS. Vancouver Scar Scale; POSAS. Patient and Observer Scar Assessment Scale

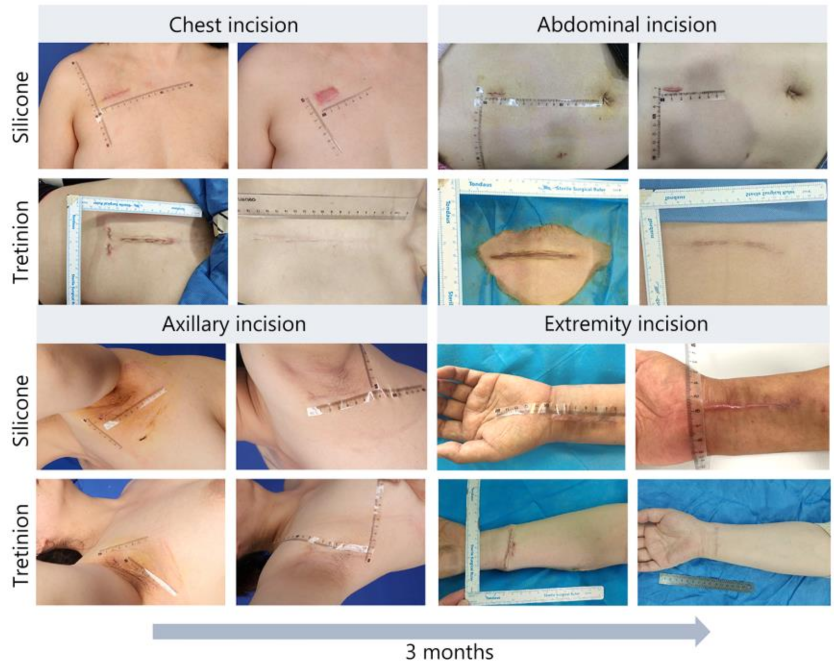

Fig. S2 Representative images of the chest, abdominal, axillary, and extremity incisions captured before and after silicone gel (Silicone) or 0.05% tretinoin cream (Tretinoin) treatment for 3 months, respectively. The images demonstrate that tretinoin cream significantly prevents HS formation and improves scar appearance. HS. Hypertrophic scar

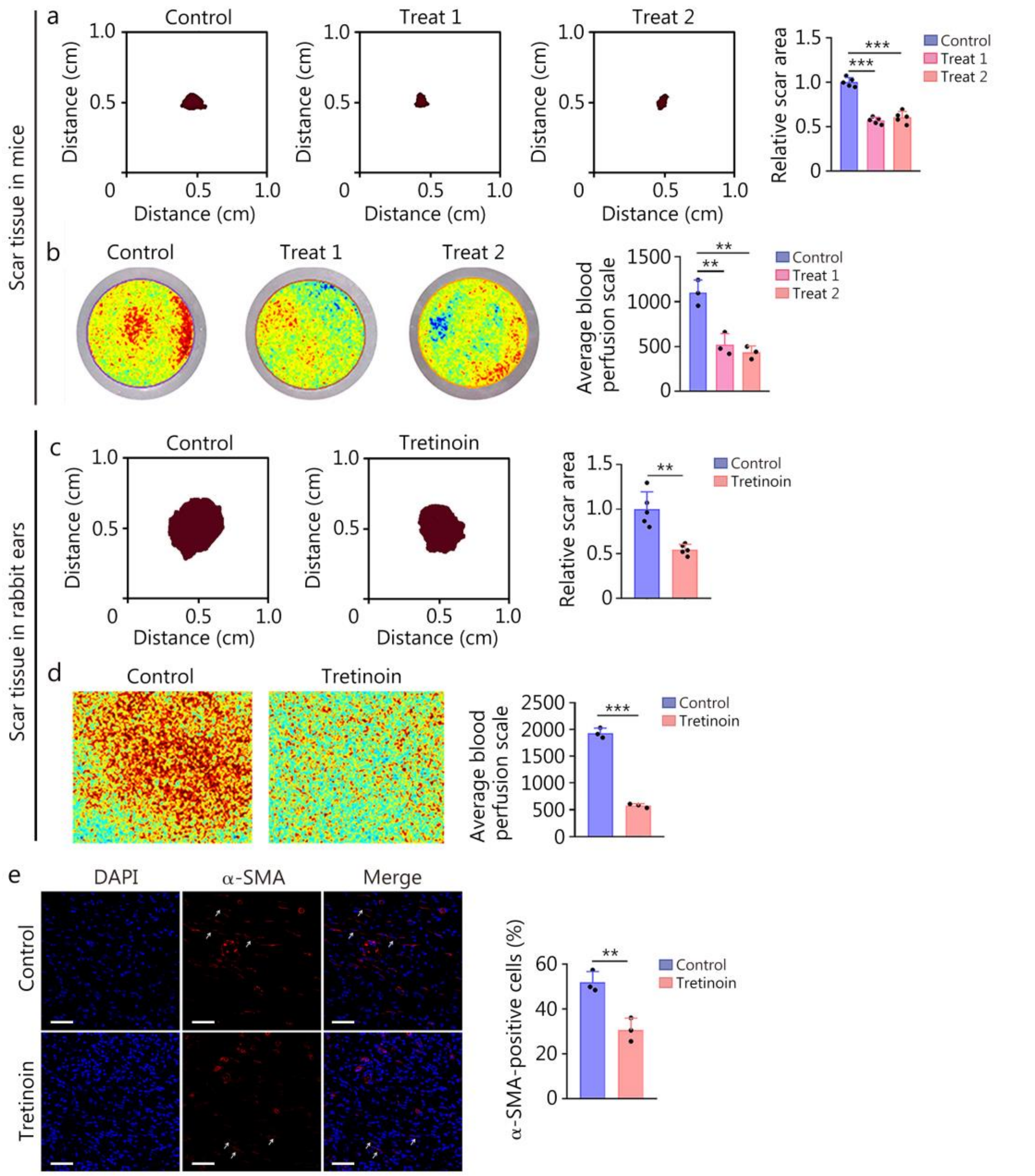

Fig. S3 Tretinoin cream reduced hypertrophic scar formation and myofibroblast activation *in vivo*. **a** A schematic representation of scar formation and the quantification of relative scar areas for completely healed wounds at 18 d after wound creation in the mice wound splinting model. PBS-treated wounds served as the Control group, while wounds treated with 0.05% tretinoin cream on day 0 and day 10 post-operation constituted the Treat 1 and Treat 2 groups, respectively (*n*=5). **b** Representative photomicrographs and quantitative analyses of blood perfusion measured by the average blood perfusion scale of scars for completely healed wounds in the Control, Treat 1, and Treat 2 groups in mice (*n*=3). **c** A schematic representation of scar formation and the quantification of relative scar areas for the hypertrophic scar at 55 d after wound construction in the rabbit ear hypertrophic scar model. PBS-treated and 0.05% tretinoin cream-treated wounds on day 21 post-operation served as the Control group and Tretinoin group, respectively (*n*=5). **d** Representative photomicrographs and quantitative analyses of blood perfusion via average blood perfusion scale of scars in the PBS (Control group) and 0.05% tretinoin cream-treated rabbit ear (Tretinoin group) at 55 d after wound construction (*n*=3). **e** Immunofluorescence staining of α-SMA for the myofibroblasts in the sections of healed wounds and quantification of the proportion of α-SMA-positive myofibroblasts with PBS treatment (Control group) and 0.05% tretinoin cream treatment group throughout the whole period (Tretinoin group) in mice. Scale bar = 200 μm. White arrows indicated the representative images of α-SMA-positive cells. $^{**}P<0.01$, $^{***}P<0.001$. PBS. Phosphate-buffered saline; α-SMA. α-smooth muscle actin

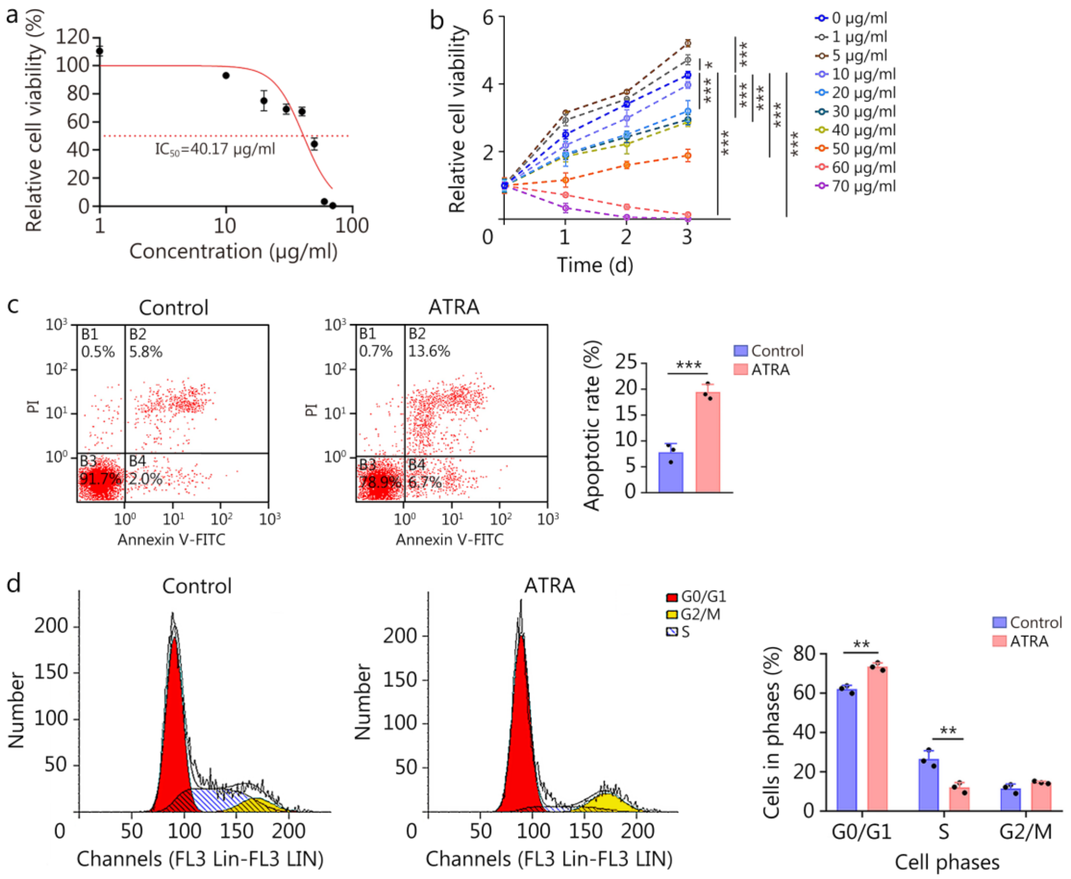

Fig. S4 Determination of optimal ATRA concentration and its effect on the cellular activity of HSFs. **a** Predicted IC_{50} values of HSFs treated with varying concentrations of ATRA for 72 h, compared to the ATRA-untreated control group, with CCK-8 assay. **b** CCK-8 assay determined the optimal concentration of ATRA for inhibiting the HSF viability as 50 μg/ml. **c** Flow cytometry analysis and quantification of proportions of apoptotic cells for both PBS (Control group) and ATRA-treated HSFs (ATRA group) (n=3). **d** Flow cytometry analysis and quantification of cell proportions at G0/G1, G2/M, and S for both PBS and ATRA-treated HSFs (n=3). $^{*}P<0.05$, $^{**}P<0.01$, $^{***}P<0.001$. ATRA. All-trans retinoic acid; HSFs. Hypertrophic scar fibroblasts; PBS. Phosphate-buffered saline; TGF-β1. Transforming growth factor-β1; α-SMA. α-smooth muscle actin; Col-1. Collagen type I; PI. Propidium Iodide; FITC. Fluorescein Isothiocyanate

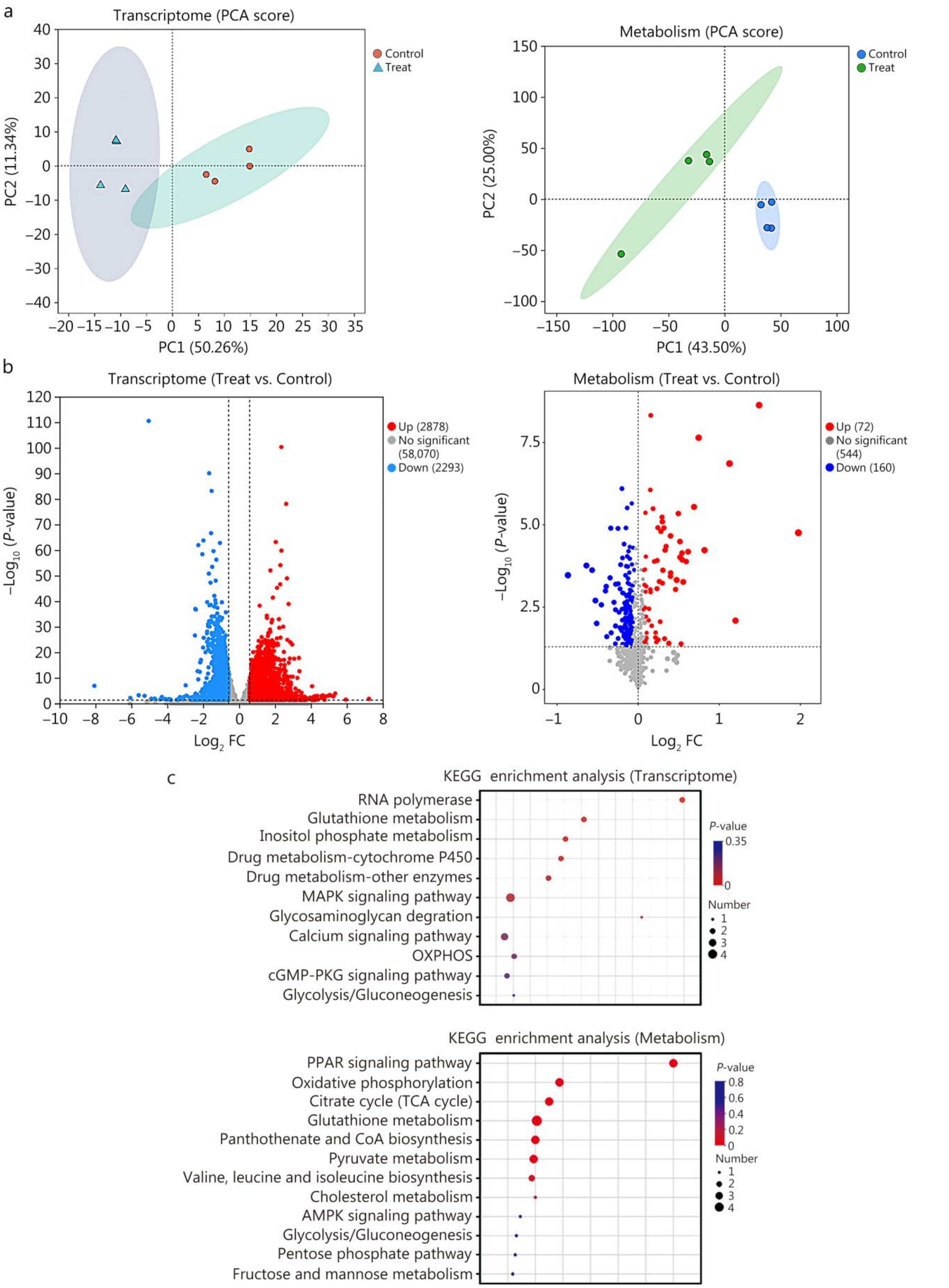

Fig. S5 Transcriptome and metabolism analysis of HSFs with and without ATRA treatment. **a** PCA based on transcriptomic (left) and metabolic (right) profiling of PBS (Control group) and ATRA-treated HSFs (Treat group). **b** Volcano plots demonstrated the differentially expressed genes (left) and metabolites (right) of PBS and ATRA-

treated HSFs. **c** Bubble plots of KEGG enrichment analysis of differential genes (top) and metabolites (bottom) of HSFs with and without ATRA treatment. HSFs. Hypertrophic scar fibroblasts; ATRA. All-trans retinoic acid; PCA. Principal component analysis; PBS. Phosphate-buffered saline; OXPHOS. Oxidative phosphorylation; MAPK. Mitogen-activated protein kinase; cGMP-PKG. Cyclic guanosine monophosphate-protein kinase G; TCA. Tricarboxylic acid cycle; AMPK. Adenosine 5‘-monophosphate (AMP)-activated protein kinase

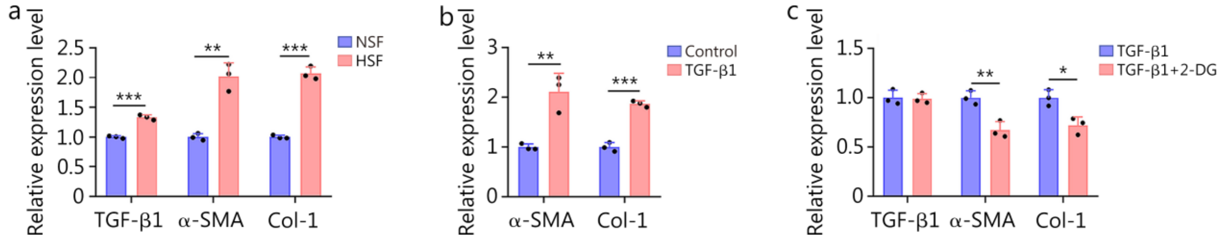

Fig. S6 Relative protein expression of TGF-β1, α-SMA, and Col-1 in NSFs and HSFs**. a** Quantification of TGF-β1, α-SMA, and Col-1 protein expression of the fibroblasts in normal skin (NSFs) and hypertrophic scar fibroblasts (HSFs) in **Fig. 3a** (*n*=3). **b** Quantification of α-SMA and Col-1 protein expression of PBS (Control group) and TGF-β1-stimulated NSFs (TGF-β1 group) in **Fig. 3h** (*n*=3). **c** Quantification of TGF-β1, α-SMA and Col-1 protein levels of NSFs treated with TGF-β1 with or without 2-DG in **Fig. 3m** (*n*=3). $^{*}P<0.05$, $^{**}P<0.01$, $^{***}P<0.001$. TGF-β1. Transforming growth factor-β1; α-SMA. α-smooth muscle actin; Col-1. Collagen type I; 2-DG. 2-Deoxy-D-glucose

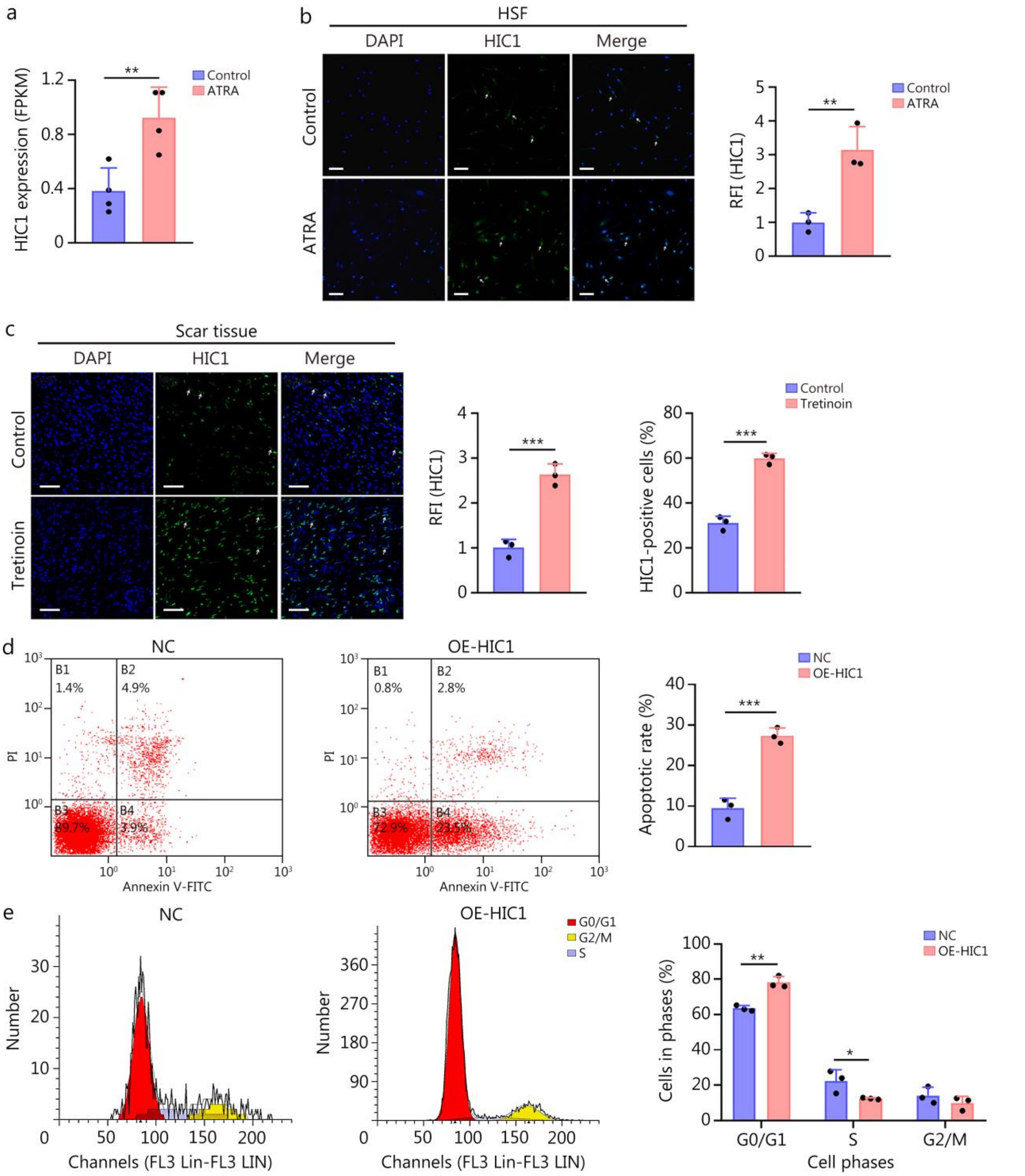

Fig. S7 ATRA inhibits HSF activation and activity by upregulating HIC1. **a** The expression of *HIC1* was quantified using FPKM for both PBS (Control group) and ATRA-treated HSFs (ATRA group) (*n*=4). **b** ICC staining of HIC1 in HSFs and quantification of the RFI of HIC1 in PBS (Control group) and ATRA-treated HSFs (ATRA group) (*n*=3). Scale bar = 100 μm. White arrows indicate the representative HIC1-positive cells. **c** IF staining for HIC1 of fibroblasts, and the relative fluorescence intensity and proportion of HIC1-positive fibroblasts were quantified in sections of healed wounds in mice treated with PBS (Control group) and 0.05% tretinoin cream (Tretinoin group) (*n*=3). Scale bar = 200 μm. White arrows indicated the representative HIC1-positive cells. **d** Flow cytometry analysis and quantification of proportions of apoptotic cells of HSFs for both the negative control group (NC group) and the HIC1 overexpression group (OE-HIC1 group) (*n*=3). **e** Flow cytometry analysis and quantification of cell proportions at G0/G1, G2/M, and S phases of HSFs in the negative control group and the HIC1 overexpression group (*n*=3). $^{*}P<0.05$, $^{**}P<0.01$, $^{***}P<0.001$. HSFs. Hypertrophic scar fibroblasts; ATRA. All-trans retinoic acid; PBS. Phosphate-buffered

saline; ICC. Immunocytochemical staining; RFI. Relative fluorescence intensity; IF. Immunofluorescence; OE. Overexpression; HIC1. Hypermethylated in cancer 1; FPKM. Fragments Per Kilobase of transcript per Million mapped reads

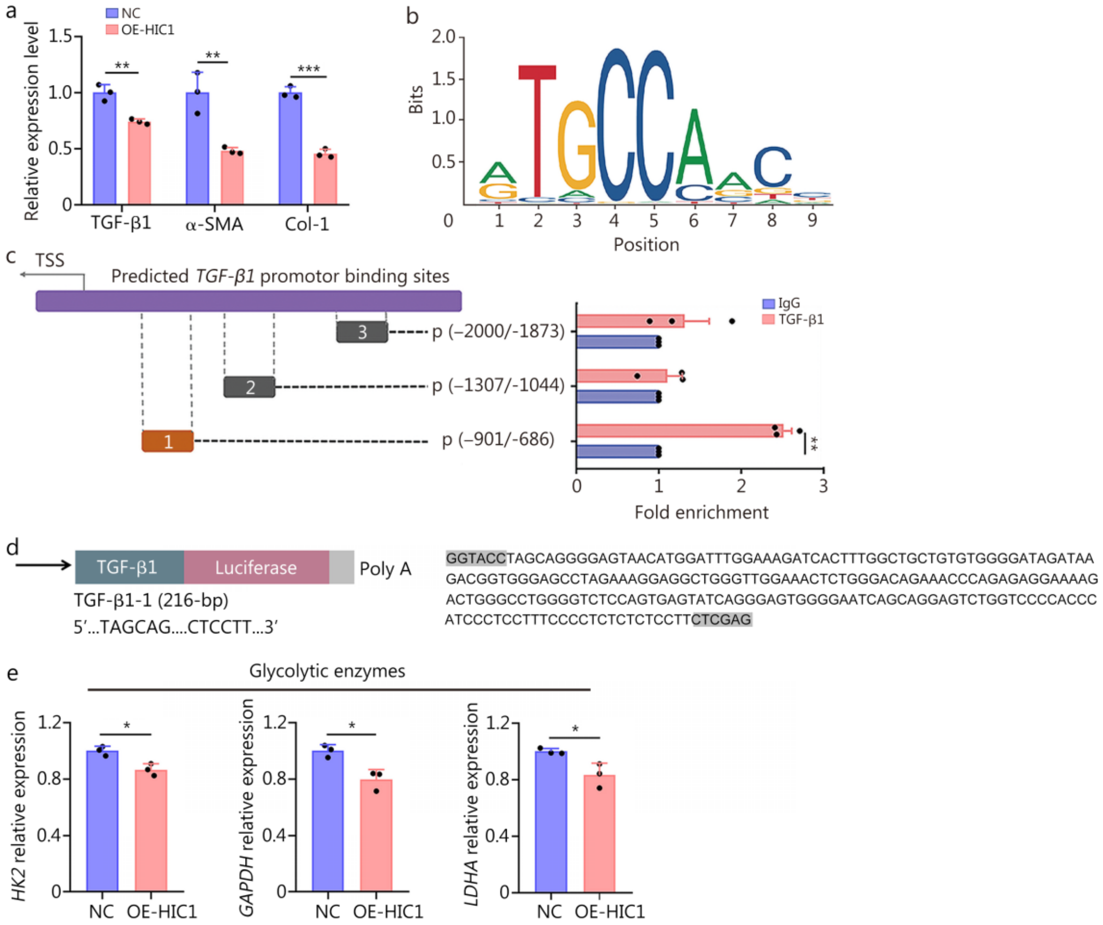

Fig. S8 Overexpression of *HIC1* in HSFs inhibits cell activation and reprograms the glycolysis process, and the results of ChIP-qPCR and firefly luciferase assays confirmed the binding of *HIC1* on *TGF-β1* gene promoter. **a** Quantification of TGF-β1, α-SMA, and Col-1 protein expression in HSFs for the negative control group (NC group) and the HIC1 overexpression group (OE-HIC1 group) in **Fig. 5j** (*n*=3). **b** The predicted binding motif of the HIC1 transcription factor obtained from JASPAR. **c** A ChIP-qPCR assay was conducted to confirm the predicted binding sites of HIC1 transcription factors on the *TGF-β1* gene promoter in HSFs, and the most significantly enriched regions of the *TGF-β1* promoter were coloured orange for constructing a plasmid. **d** A schematic of TGF-β1 firefly luciferase reporters. The 216-bp DNA sequence of TGF-β1 is located between the KpnI and XhoI restriction sites upstream of the stop codon of the firefly luciferase gene within a PGL3 basic vector. The cloned DNA sequences of the *TGF-β1* promoter within the reporter vector are shown with cloning sites highlighted in grey. **e** Differential mRNA expression levels of glycolytic enzymes in HSFs in the NC group and the OE-HIC1 group detected by RT-qPCR (*n*=3). $^{*}P<0.05$, $^{**}P<0.01$, $^{***}P<0.001$. OE. Overexpression; HIC1. Hypermethylated in cancer 1; HSFs. Hypertrophic scar fibroblasts; TGF-β1. Transforming growth factor-β1; α-SMA. α-smooth muscle actin; Col-1. Collagen type I; ChIP. Chromatin immunoprecipitation; RT-qPCR. Quantitative reverse transcription PCR; TSS. Transcription start sites

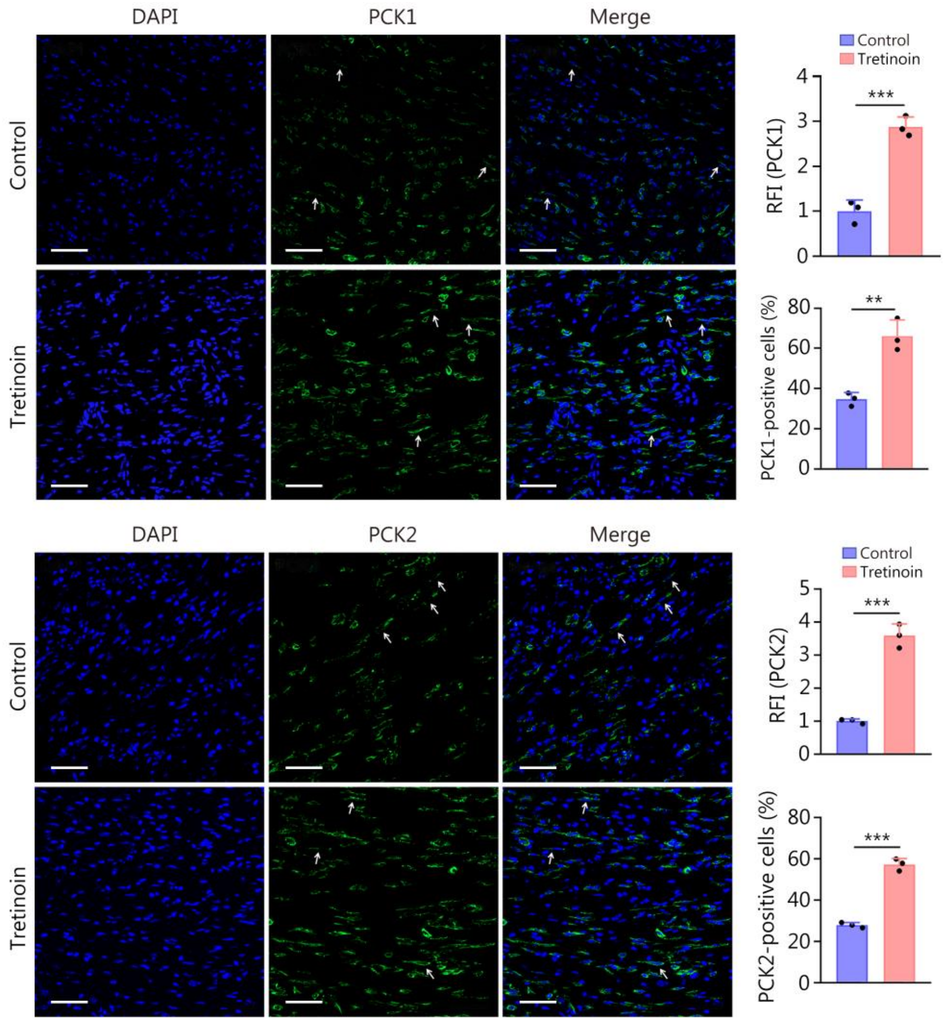

Fig. S9 Validation of PCK1 and PCK2 expression *in vivo*. IF staining of PCK1 (top) and PCK2 (bottom), as well as the quantification of the PCK1 and PCK2 relative fluorescence intensity, and proportion of PCK1/PCK2-positive fibroblasts in sections of healed wounds in mice from the Control and Tretinoin treatment groups (*n*=3). Scale bar = 200 μm. White arrows indicated the representative PCK1-postive or PCK2-positive cells. $^{*}P$<0.05, $^{**}P$<0.01, $^{***}P$<0.001. PCK1. Phosphoenolpyruvate carboxykinase 1; PCK2. Phosphoenolpyruvate carboxykinase 2; IF. Immunofluorescence staining; DAPI. 4',6-Diamidino-2-phenylindole; RFI. Relative Fluorescence Intensity

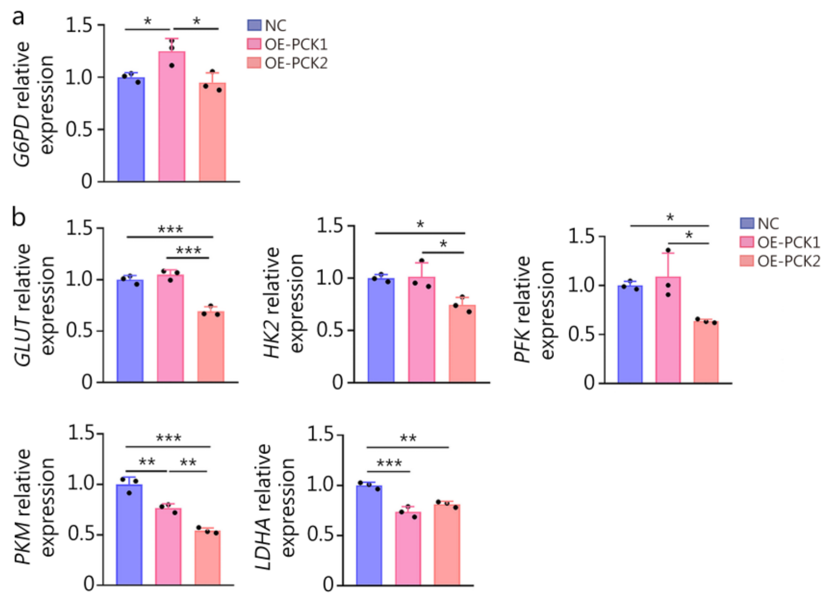

Fig. S10 Overexpression of *PCK1* and *PCK2* in HSFs regulates the expression of gluconeogenic (**a**) and glycolytic enzymes (**b**). Differential mRNA expression levels of gluconeogenic and glycolytic enzymes in HSFs from the three experimental groups: the negative control (NC group), *PCK1* overexpression (OE-PCK1 group), and *PCK2* overexpression (OE-PCK2 group), detected by RT-qPCR analysis (n=3). $^{*}P$<0.05, $^{**}P$<0.01, $^{***}P$<0.001. OE. Overexpression; HIC1. Hypermethylated in cancer 1; HSFs. Hypertrophic scar fibroblasts; PCK1. Phosphoenolpyruvate carboxykinase 1; PCK2. Phosphoenolpyruvate carboxykinase 2; RT-qPCR. Quantitative reverse transcription PCR

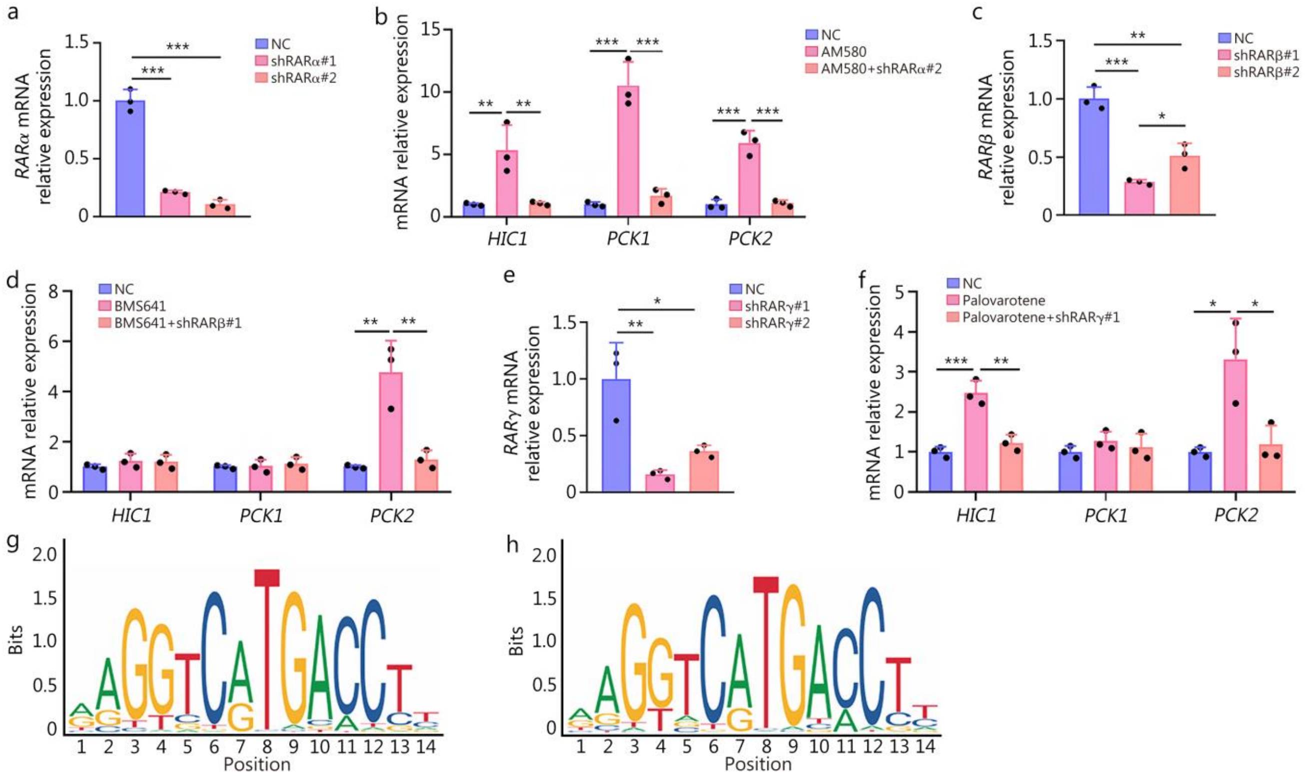

Fig. S11 The potential regulatory patterns of RARs on HIC1, PCK1, and PCK2. **a** The knockdown efficiency of *RARα* by two RARα shRNAs in HSFs was determined by RT-qPCR (*n*=3). **b** Relative expression levels of *HIC1*, *PCK1*, and *PCK2* in HSFs within the negative control group (NC group), AM580-treated group (AM580 group), and AM580-treated with subsequent *RARα* knockdown group (AM580+shRAR*α*#2 group) (*n*=3). **c** The knockdown efficiency of *RARβ* by two RARβ shRNAs in HSFs was determined by RT-qPCR (*n*=3). **d** Relative expressions of *HIC1*, *PCK1*, and *PCK2* in HSFs within the negative control group, BMS641-treated group (BMS641 group) and BMS641-treated with subsequent *RARβ* knockdown group (BMS641+shRAR*β*#1 group). **e** The knockdown efficiency of *RARγ* by two RARγ shRNAs in HSFs was determined by RT-qPCR. **f** Relative expressions of *HIC1*, *PCK1*, and *PCK2* in HSFs within the negative control group, Palovarotene-treated group (Palovarotene group), and Palovarotene-treated with subsequent *RARγ* knockdown group (Palovarotene+shRARγ#1 group). **g** The predicted RARβ transcription factor binding motif and their binding potentials with HIC1 and PCK2 obtained from JASPAR (*n*=3). **h** The predicted RARγ transcription factor binding motif and their binding potentials with HIC1 and PCK2 obtained from JASPAR (*n*=3). $^{*}P<0.05$, $^{**}P<0.01$, $^{***}P<0.001$. RARs. Retinoic acid receptors; HIC1. Hypermethylated in cancer 1; HSFs. Hypertrophic scar fibroblasts; PCK1. Phosphoenolpyruvate carboxykinase 1; PCK2. Phosphoenolpyruvate carboxykinase 2; RARα. Retinoic acid receptor alpha; RARβ. Retinoic acid receptor beta; RARγ. Retinoic acid receptor gamma; RT-qPCR. Quantitative reverse transcription PCR

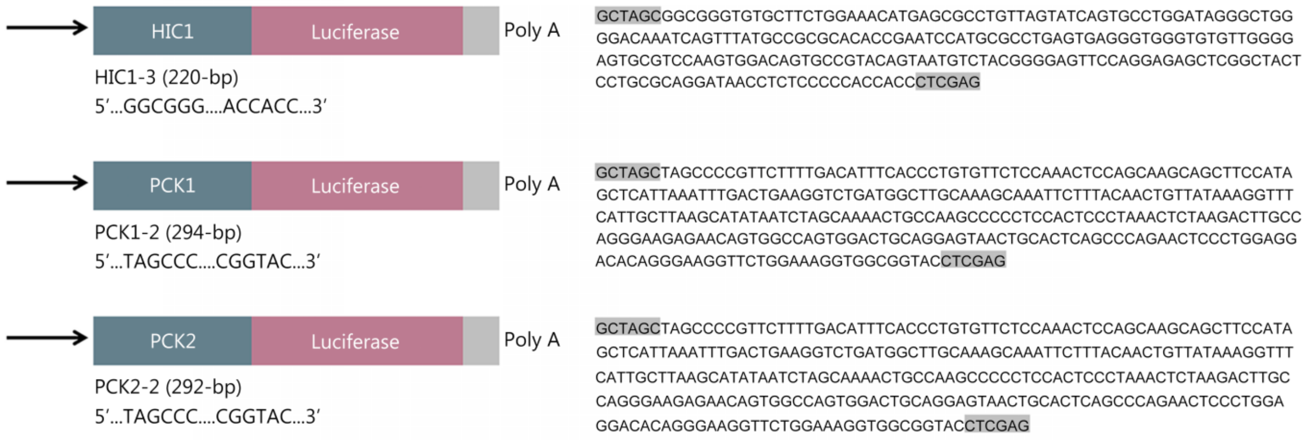

Fig. S12 Schematic diagrams of firefly luciferase reporters. Schematics of *HIC1*, *PCK1*, and *PCK2* firefly luciferase reporters. A 220-bp DNA sequence of *HIC1*, a 294-bp DNA sequence of *PCK1*, and a 292-bp DNA sequence of *PCK2* were individually inserted between the KpnI and XhoI restriction sites upstream of the stop codon of the firefly luciferase gene within a PGL3 basic vector, generating the HIC1, PCK1, and PCK2 constructs, respectively. The cloned DNA sequences of *HIC1*, *PCK1*, and *PCK2* promoters within the reporter vector, with cloning sites highlighted in grey. HIC1. Hypermethylated in cancer 1; PCK1. Phosphoenolpyruvate carboxykinase 1; PCK2. Phosphoenolpyruvate carboxykinase 2

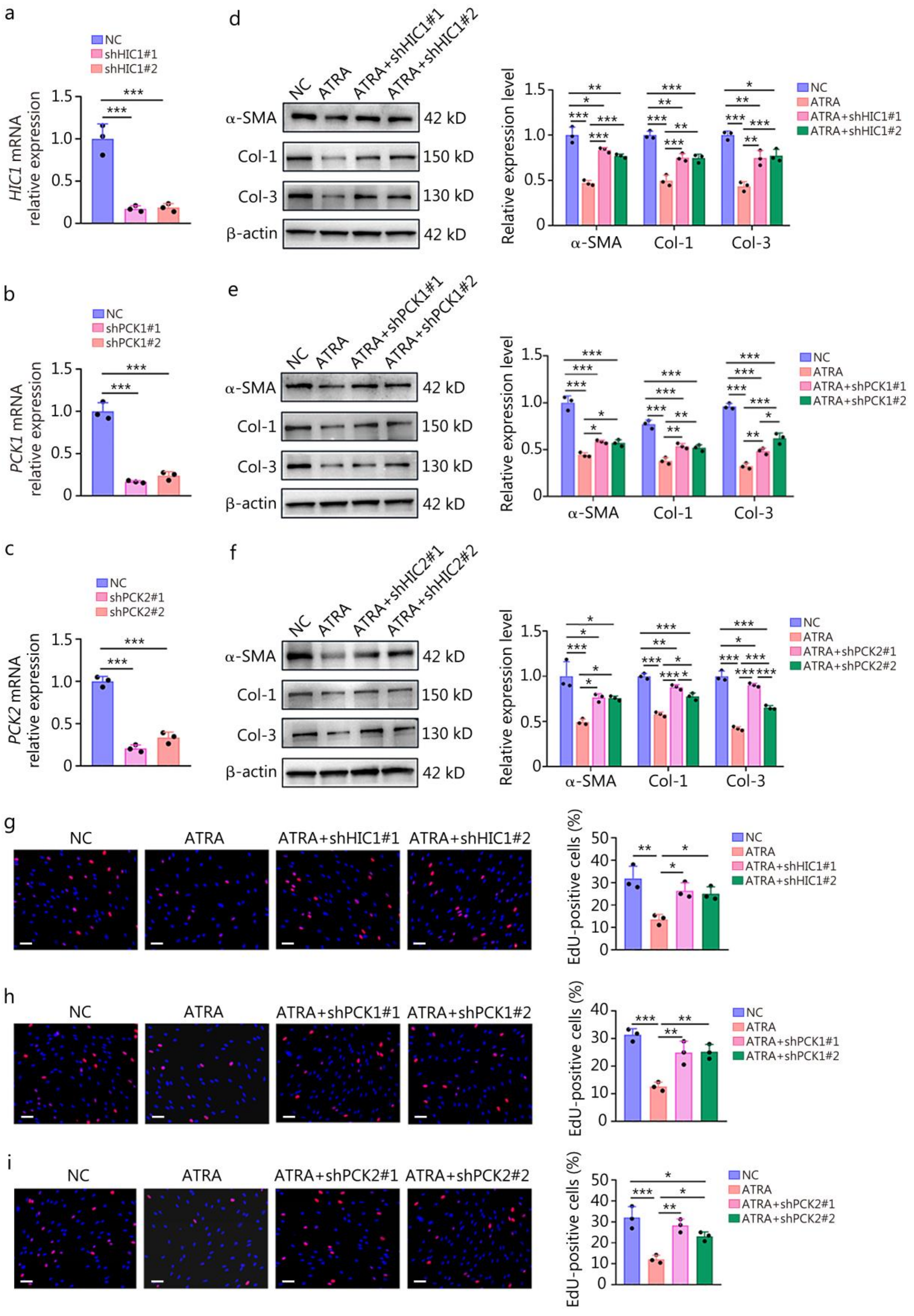

Fig. S13 The shRNAs rescue experiments for HSFs treated with ATRA. **a-c** The knockdown efficiency of two distinct shRNAs designed for *HIC1* (**a**), *PCK1* (**b**), and *PCK2* (**c**) in HSFs evaluated by RT-qPCR (*n*=3). **d** Western blotting assay and quantification of the expression levels of α-SMA, Col-1, and Col-3 protein in HSFs in the negative control group (NC group), ATRA-treated group (ATRA group), and the groups treated with ATRA followed by *HIC1* knockdown (ATRA+shHIC1#1/shHIC1#2 group) (*n*=3). **e** Western blotting assay and quantification of the expression

levels of α-SMA, Col-1, and Col-3 protein in HSFs in the negative control group (NC group), ATRA-treated group (ATRA group), and the groups treated with ATRA followed by *PCK1* knockdown (ATRA+shPCK1#1/shPCK1#2 group) (*n*=3). **f** Western blotting assay and quantification of the expression levels of α-SMA, Col-1, and Col-3 protein in HSFs in the negative control group (NC group), ATRA-treated group (ATRA group), and the groups treated with ATRA followed by *PCK2* knockdown (ATRA+shPCK2#1/shPCK2#2 group) (*n*=3). **g** Representative images of EdU staining and the proportion quantification of the EdU-positive HSFs in the NC group, ATRA group, and ATRA+shHIC1#1/shHIC1#2 groups (*n*=3). Scale bar=100 μm. **h** Representative images of EdU staining and the proportion quantification of the EdU-positive HSFs in the NC group, ATRA group, and ATRA+shPCK1#1/shPCK1#2 groups (*n*=3). Scale bar=100 μm. **i** Representative images of EdU staining and the proportion quantification of the EdU-positive HSFs in the NC group, ATRA group, and ATRA+shPCK2#1/shPCK2#2 groups (*n*=3). Scale bar=100 μm. HSFs. Hypertrophic scar fibroblasts; HIC1. Hypermethylated in cancer 1; PCK1. Phosphoenolpyruvate carboxykinase 1; PCK2. Phosphoenolpyruvate carboxykinase 2; ATRA. All-trans retinoic acid; RT-qPCR. Quantitative reverse transcription PCR

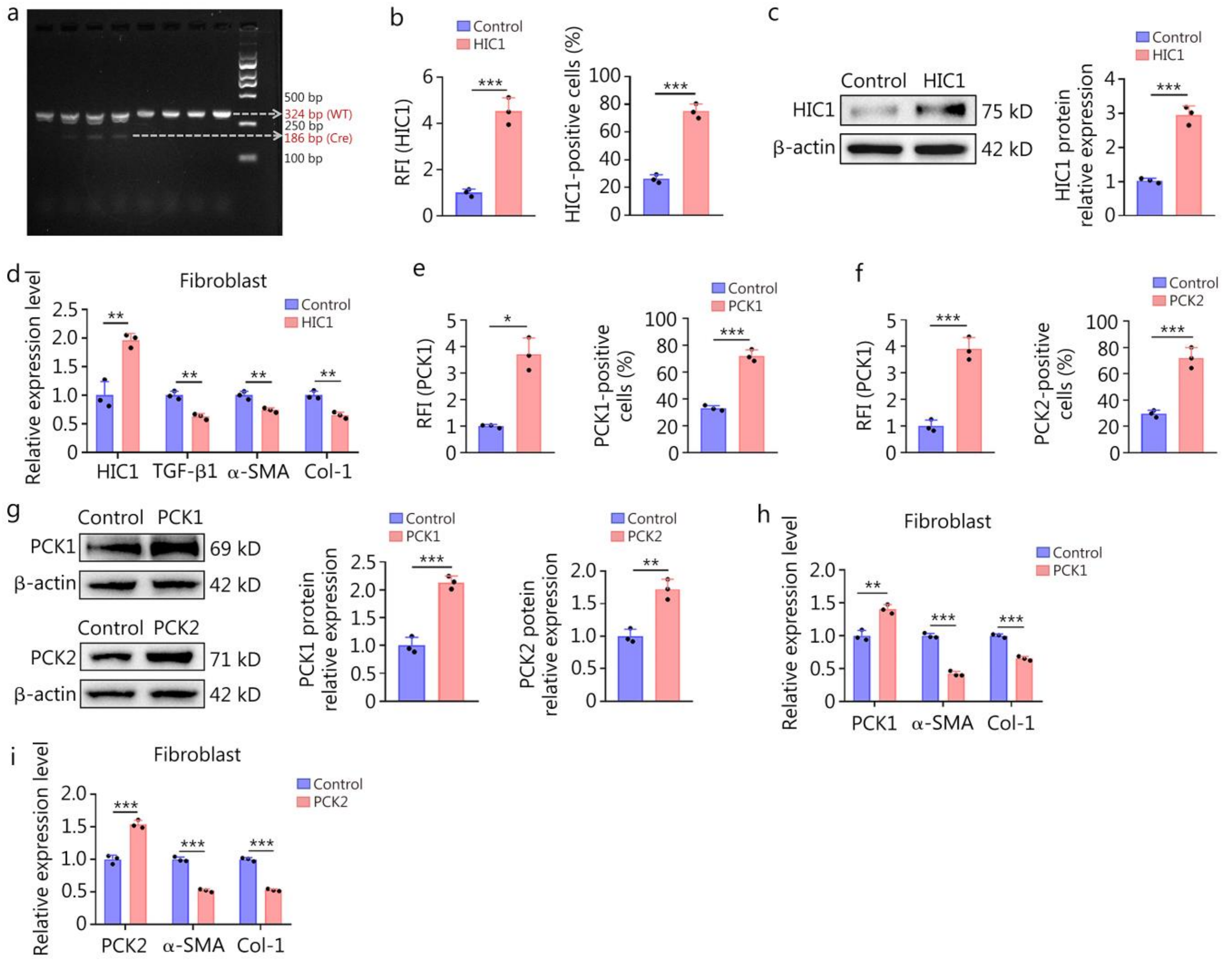

Fig. S14 Fibroblast-specific *HIC1*, *PCK1*, or *PCK2* overexpression in wounds alleviated scar formation in Col1a2-CreER mice**. a** The Col1a2-CreER mice were identified by PCR with RNA-specific product (186 bp). **b** Quantification of the relative fluorescence intensity (RFI) of HIC1 and proportion of HIC1 positive fibroblasts of sections from healed wounds in the AAV-HIC1 group (HIC1 group) compared with the AAV-control group (Control group) in Col1a2-CreER mice in **Fig. 8c** (*n*=3). **c** Western blotting assay and quantification of HIC1 protein levels in healed wound tissues of Col1a2-CreER mice in the control and HIC1 groups (*n*=3). **d** Quantification of HIC1, TGF-β1, α-SMA, and Col-1 protein expression in fibroblasts isolated from the regenerated skin of the healed wounds of mice in the control and HIC1 groups in **Fig. 8d** (*n*=3)**. e, f** Quantification of the relative fluorescence intensity (RFI) of PCK1 and PCK2, and proportions of PCK1 and PCK2 positive fibroblasts of sections from healed wounds in the AAV-PCK1 group (PCK1 group) (**e**), and AAV-PCK2 group (PCK2 group) (**f**), compared with the AAV-control group (Control group) in Col1a2-CreER mice in **Fig. 8e** (*n*=3). **g** Western blotting assay and quantification of PCK1 and PCK2 protein levels in healed wound tissues of Col1a2-CreER mice from the control, PCK1, and PCK2 groups (*n*=3). **h** Quantification of PCK1, α-SMA, and Col-1 protein expression in fibroblasts isolated from the regenerated skin from the healed wounds of mice in the control and PCK1 groups in **Fig. 8f** (*n*=3). **i** Quantification of PCK2, α-SMA, and Col-1 protein expression in fibroblasts isolated from the regenerated skin from the healed wounds of mice in the control and PCK2 groups in **Fig. 8f** (*n*=3). AAV-HIC1. AAV-CMV-loxP-stop-loxP-HIC; AAV-PCK1. AAV-CMV-loxP-stop-loxP-PCK1; AAV-PCK2. AAV-CMV-loxP-stop-loxP-PCK2; AAV-control. AAV-CMV-loxP-stop-loxP; HIC1. Hypermethylated in cancer 1; PCK1. Phosphoenolpyruvate carboxykinase 1; PCK2. Phosphoenolpyruvate carboxykinase 2; α-SMA. α-smooth muscle actin; Col-1. Collagen type I; WT. Wild-type

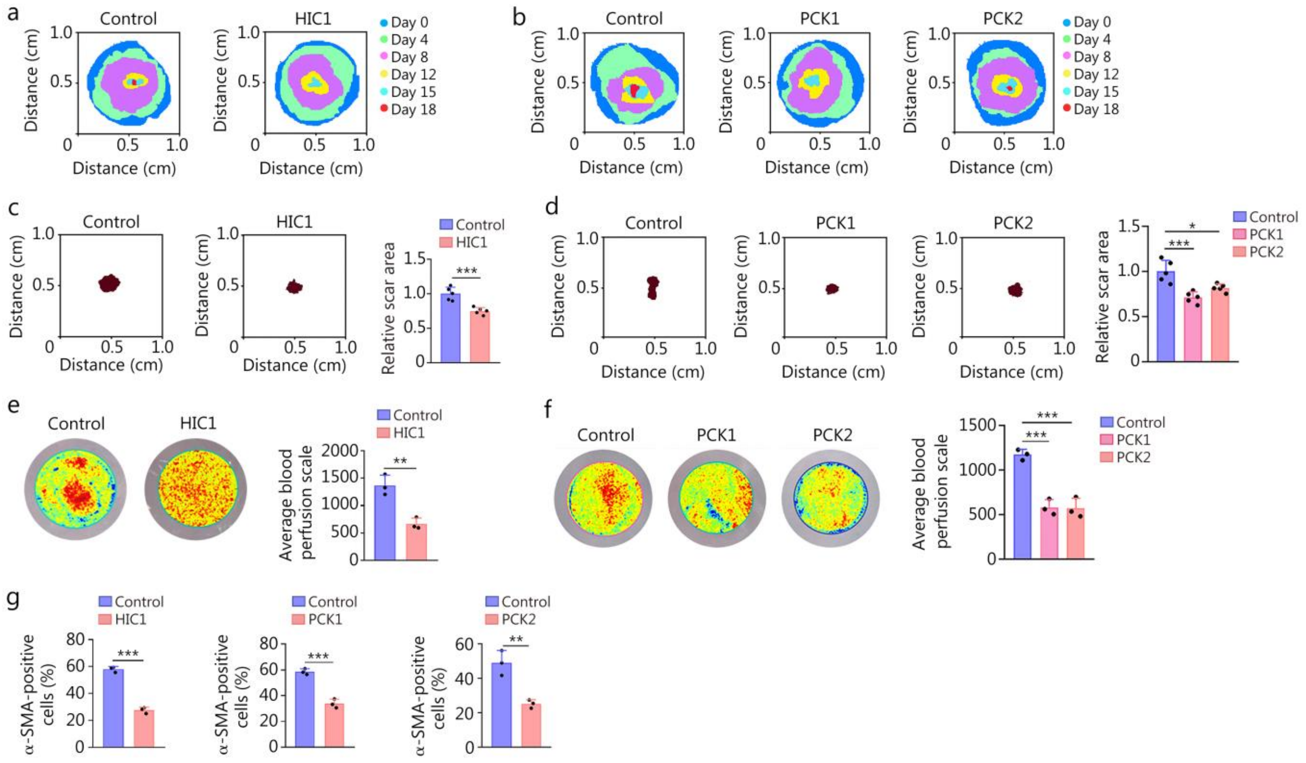

Fig. S15 Fibroblast-specific *PCK1* and *PCK2* overexpression in wounds alleviated scar formation in Col1a2-CreER mice. **a** Schematics of wound healing processes of Col1a2-CreER mice in control and HIC1 groups. **b** Schematics of wound healing processes of Col1a2-CreER mice in control, PCK1, and PCK2 groups. **c** A schematic of scar formation and the quantification of scar areas in the HIC1 group relative to the scar areas in the control group for completely healed wounds at 18 d after wound construction (*n*=5). **d** Schematics of scar formation and the quantification of scar areas in PCK1 and PCK2 groups relative to the scar areas in the control group for completely healed wounds at 18 days after wound creation (*n*=5). **e** Representative photomicrographs and quantitative analyses of blood perfusion measured by the average blood perfusion scale of scars for completely healed wounds in the control and HIC1 groups in mice. **f** Representative photomicrographs and quantitative analyses of blood perfusion, measured by the average blood perfusion scale of scars for completely healed wounds in mice from the control, PCK1, and PCK2 groups (*n*=3). **g** Quantification of the proportions of α-SMA-positive myofibroblasts in the healed wound tissues of Col1a2-CreER mice in control, HIC1, PCK1, and PCK2 groups in **Fig. 8i, j** (*n*=3). RFI. Relative fluorescence intensity; PCK1. Phosphoenolpyruvate carboxykinase 1; PCK2. Phosphoenolpyruvate carboxykinase 2; α-SMA. α-smooth muscle actin

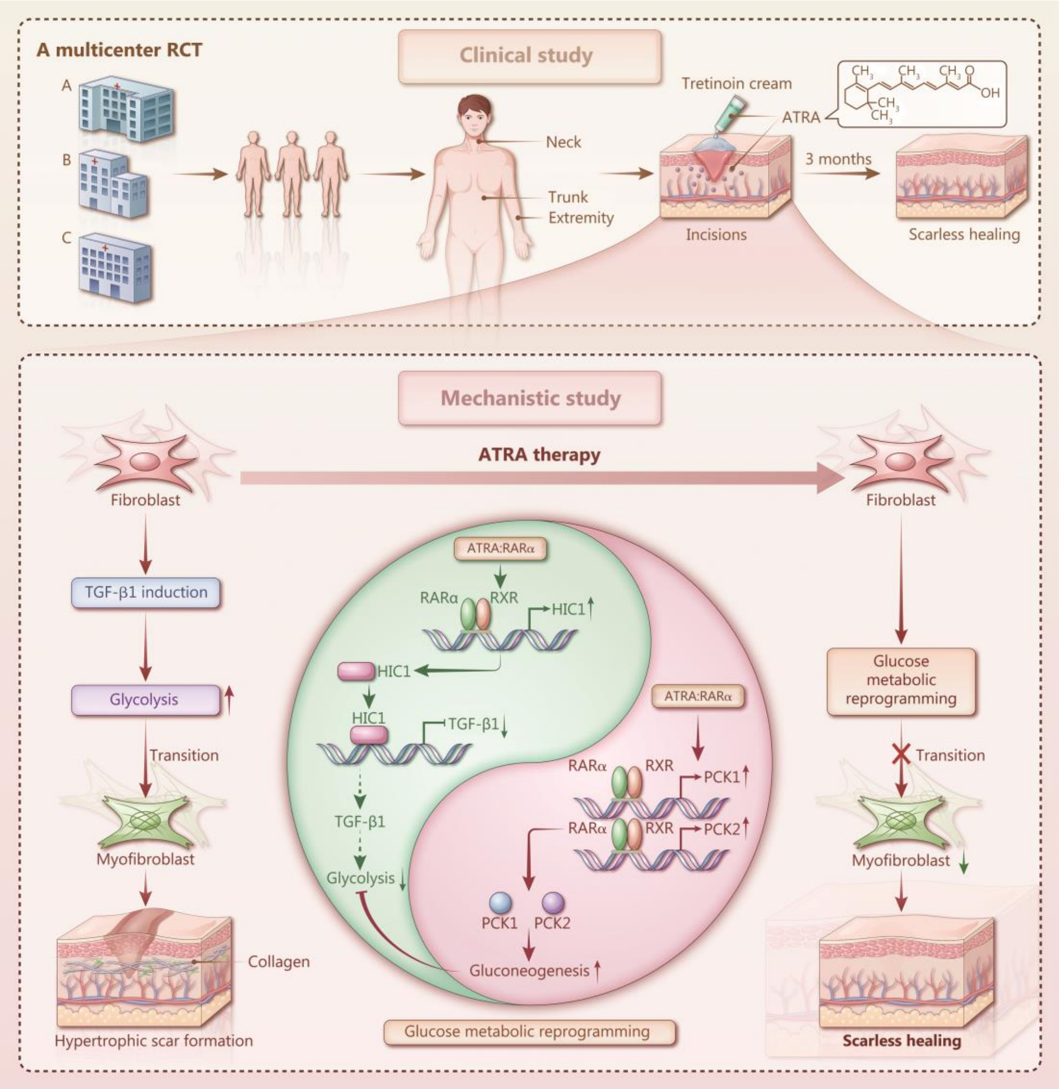

Fig. S16 Summary of the key findings in this paper. The schematic is divided into two segments: the upper segment illustrates the study strategies based on the multicenter RCT aimed at evaluating the efficacy of tretinoin cream in preventing postoperative HS formation. The lower segment mainly focuses on the mechanistic study, elucidating that enhanced aerobic glycolysis in HSFs drives HS pathogenesis; on this basis, this work further reveals that ATRA reprograms cellular glucose metabolism via HIC1, PCK1, and PCK2, thereby inhibiting the proliferation and activation of HSFs and reversing the fibrotic phenotype of HS through a novel mechanism. RCT. Randomized controlled trial; HS. Hypertrophic scar; ATRA. All-trans retinoic acid; HSFs. Hypertrophic scar fibroblasts; HIC1. Hypermethylated in cancer 1; PCK1. Phosphoenolpyruvate carboxykinase 1; PCK2. Phosphoenolpyruvate carboxykinase 2; TGF-β1. Transforming growth factor-β1; RXR. Retinoid X receptor
